# Immunopeptidome of hepatocytes isolated from patients with HBV infection and hepatocellular carcinoma

**DOI:** 10.1016/j.jhepr.2022.100576

**Published:** 2022-09-05

**Authors:** Monique T.A. de Beijer, Karel Bezstarosti, Robbie Luijten, Wouter A.S. Doff, Patrick P.C. Boor, Roel F.A. Pieterman, Rachid Bouzid, Paula J. Biesta, Jan N.M. Ijzermans, Michail Doukas, Robert A. de Man, Andrea M. Woltman, Jeroen A.A. Demmers, Sonja I. Buschow

**Affiliations:** 1 Department of Gastroenterology and Hepatology, Erasmus MC University Medical Center Rotterdam, the Netherlands; 2 Proteomics Center, Erasmus MC, Rotterdam, the Netherlands; 3 Department of Surgery, Erasmus MC, Rotterdam, the Netherlands; 4 Department of Pathology, Erasmus MC, Rotterdam, the Netherlands

**Keywords:** Antigen presentation, Liver cancer, T cell epitope, HLA, MHC, Peptidome, Cancer testis antigen, Cancer germline antigen, Viral hepatitis, cHBV, chronic HBV, DDA, data-dependent acquisition, GO, Gene Ontology, HBV, Hepatitis B virus, HCC, hepatocellular carcinoma, HLA, human leucocyte antigen, IEDB, Immune Epitope Database, IFNγ, interferon γ, IP, immunoprecipitation, KEGG, Kyoto Encyclopedia of Genes and Genomes, LSEC, liver sinusoidal cell, MS, mass spectrometry, PBMCs, peripheral blood mononuclear cells, Pol, polymerase, PRM, parallel reaction monitoring, TAA, tumour-associated antigen

## Abstract

**Background & Aims:**

Antigen-specific immunotherapy is a promising strategy to treat HBV infection and hepatocellular carcinoma (HCC). To facilitate killing of malignant and/or infected hepatocytes, it is vital to know which T cell targets are presented by human leucocyte antigen (HLA)-I complexes on patient-derived hepatocytes. Here, we aimed to reveal the hepatocyte-specific HLA-I peptidome with emphasis on peptides derived from HBV proteins and tumour-associated antigens (TAA) to guide development of antigen-specific immunotherapy.

**Methods:**

Primary human hepatocytes were isolated with high purity from (HBV-infected) non-tumour and HCC tissues using a newly designed perfusion-free procedure. Hepatocyte-derived HLA-bound peptides were identified by unbiased mass spectrometry (MS), after which source proteins were subjected to Gene Ontology and pathway analysis. HBV antigen and TAA-derived HLA peptides were searched for using targeted MS, and a selection of peptides was tested for immunogenicity.

**Results:**

Using unbiased data-dependent acquisition (DDA), we acquired a high-quality HLA-I peptidome of 2 × 10^5^ peptides that contained 8 HBV-derived peptides and 14 peptides from 8 known HCC-associated TAA that were exclusive to tumours. Of these, 3 HBV- and 12 TAA-derived HLA peptides were detected by targeted MS in the sample they were originally identified in by DDA. Moreover, 2 HBV- and 2 TAA-derived HLA peptides were detected in samples in which no identification was made using unbiased MS. Finally, immunogenicity was demonstrated for 5 HBV-derived and 3 TAA-derived peptides.

**Conclusions:**

We present a first HLA-I immunopeptidome of isolated primary human hepatocytes, devoid of immune cells. Identified HBV-derived and TAA-derived peptides directly aid development of antigen-specific immunotherapy for chronic HBV infection and HCC. The described methodology can also be applied to personalise immunotherapeutic treatment of liver diseases in general.

**Lay summary:**

Immunotherapy that aims to induce immune responses against a virus or tumour is a promising novel treatment option to treat chronic HBV infection and liver cancer. For the design of successful therapy, it is essential to know which fragments (*i.e.* peptides) of virus-derived and tumour-specific proteins are presented to the T cells of the immune system by diseased liver cells and are thus good targets for immunotherapy. Here, we have isolated liver cells from patients who have chronic HBV infection and/or liver cancer, analysed what peptides are presented by these cells, and assessed which peptides are able to drive immune responses.

## Highlights

• We developed a perfusion-free method to isolate primary hepatocytes that are depleted of immune cells.
• We derived a large-scale unbiased hepatocyte HLA ligandome from patients with HBV and/or HCC.
• The ligandome included peptides derived from HBV proteins and tumour-associated antigens (TAA).
• Using a targeted MS regime, the detection sensitivity of several HBV and TAA-derived peptides could be increased.
• Immunogenicity was demonstrated for a selection of TAA- and HBV-derived HLA peptides.

## Graphical abstract

**Figure:**
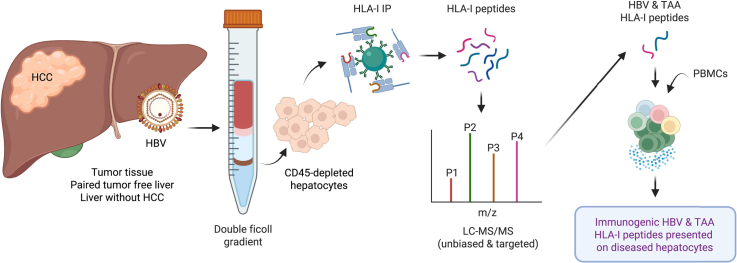

## Introduction

Antigen-specific immunotherapy has emerged as a promising treatment strategy for chronic HBV (cHBV) and hepatocellular carcinoma (HCC). Considerable efforts have been made to identify HCC-associated antigens via genome, transcriptome, and/or proteome analysis.[1], [2], [3], [4], [5], [6], [7], [8] These studies contributed to the identification of T cell epitopes that can be used in immunotherapy to target HCC.^9^ Like for HCC, several HBV-derived epitopes have been identified.[9], [10], [11] HBV antigens are of interest to cure cHBV and might also be useful targets in the treatment of HBV-related HCC, as HBV can integrate in the host genome, after which integrated HBV DNA is still present in HCC tissues.[12], [13], [14] However, for both tumour-associated antigens (TAA) and HBV-derived antigens, epitope identification mostly relies on the detection and characterisation of cognate T cells, whereas it remains largely unknown whether these T cells can recognise their cognate epitope on diseased target cells. This vital question can now be addressed using mass spectrometry (MS)-based HLA immunopeptidomics.^15^ Using unbiased MS (or data-dependent acquisition [DDA]), the HLA peptidome can be identified without *a priori* knowledge of peptide identity by mapping mass spectra to a predefined protein sequence database. Hence, this technique is extremely useful when aiming to identify previously unknown HLA-binding peptides. In contrast, targeted MS (*i.e.* parallel reaction monitoring [PRM]) allows for the detection of preselected HLA peptides of interest. MS-based immunopeptidomic approaches are already applied in the context of several diseases[16], [17], [18] including HCC.^3^^,^^7^ However, current studies mostly report HLA peptidomes of whole tissue that also contain confounding peptidomes of infiltrating leucocytes that express far superior levels of HLA-I compared with hepatocytes.^19^ Thus, only the specific peptidome of isolated diseased target cells can reveal HLA peptides that render these cells CD8^+^ T cell targets.^20^ Here, we aim to uncover the hepatocyte-specific HLA-I peptidome with emphasis on peptides derived from HBV proteins and TAAs to guide the development of effective antigen-specific immunotherapy.

## Materials and methods

A more detailed description of applied methods, used reagents, and patient materials is described in the Supplementary information.

### Peptide identification and analysis

MS/MS spectra obtained by DDA (Peaks Studio v10.5, Bioinformatics Solutions Inc., Waterloo, USA) were searched against a composite database of sequences downloaded from Uniprot (for *H. sapiens* [version August 2019], *S. aureus*, and HDV) and HBVdb^21^ (for HBV; searched entries: 190,398; available from https://hbvdb.ibcp.fr/HBVdb/). The peptide false discovery rate was set to 0.05, and output from *de novo* analysis was not analysed further. Peptides with a length of 9–11 amino acids were mapped to HLA using NetMHCpan4.1^22^ and identified as binders when rank ≤2.0%. Positioning of peptide sequences within source proteins was determined by alignment with the HBV reference sequence NC_003977.2 (HBV peptides) or the Swiss-Prot canonical protein sequence (for TAA)^23^ using COBALT.^24^ The novelty of identified peptides was assessed using the Immune Epitope Database (IEDB)^9^ last accessed on 12 March 2021.

### Proteins enriched in liver and expression in normal tissues

A list of proteins enriched in liver (n = 936) was downloaded from the Human Protein Atlas v19.3,^25^ available at http://v19.proteinatlas.org. All HLA peptides from these source proteins were extracted, after which the percentage of presented liver-enriched proteins as part of the total number of presented proteins within each tissue type was calculated. The percentage of liver-enriched proteins within the human genome was calculated as ‘(936/20,999) × 100% = 4.5%,’ as the number of protein-coding genes in the human genome is 20,999 according to GeneCards v4.14, available at https://www.genecards.org/.

### Protein expression in healthy tissues

Protein expression data from GeneCards (MS-based proteomics)^26^ and the Human Protein Atlas (immunohistochemistry on tissue micro arrays) were used to define novel tumour-associated proteins that are not expressed in healthy tissues other than testis. For this purpose, we additionally aligned all peptide sequences from candidate novel tumour-associated peptides against the human genome using the UniProt Swiss-Prot Peptide search.^23^

### Immunogenicity

Peripheral blood mononuclear cells (PBMCs) were stimulated at 1 × 10^6^/ml with a pool containing a maximum of 6 peptides of interest (Peptide2.0; >90% pure) at a concentration of 10 μg/ml/peptide. After 14 days 200,000 cultured PBMCs were restimulated in duplicate for 48 h with each peptide of interest separately or corresponding controls. Interferon γ (IFNγ) production was assessed by subjecting supernatants of restimulation cultures to the ELISA Max Standard Set Human IFNγ (BioLegend, San Diego, USA) according to the manufacturer’s instructions. Absorbance was read at 450 nm using an Infinite 200 PRO microplate reader (Tecan, Männedorf, Switzerland).

### Data visualisation and statistical analysis

The graphical abstract was created using Biorender.com. Euler diagrams were made using nVenn,^27^ available at http://degradome.uniovi.es/cgi-bin/nVenn/nvenn.cgi. Additional data analysis and visualisation were performed using GraphPad Prism 8 (GraphPad Software Inc, San Diego, USA), Illustrator v25.2 (Adobe, San Jose, USA), and RStudio v1.0.44 (RStudio, Boston, USA). Usage of statistical tests is indicated in the figure legends and based on whether the samples tested are paired and whether data are normally distributed.

### Patient and public involvement

Patient advisory groups or individual patients did not contribute to study design, data interpretation, or writing/editing of the results.

## Results

### Primary hepatocyte isolation yields high quantities of pure HLA-I-expressing hepatocytes

**Fig. 1:**
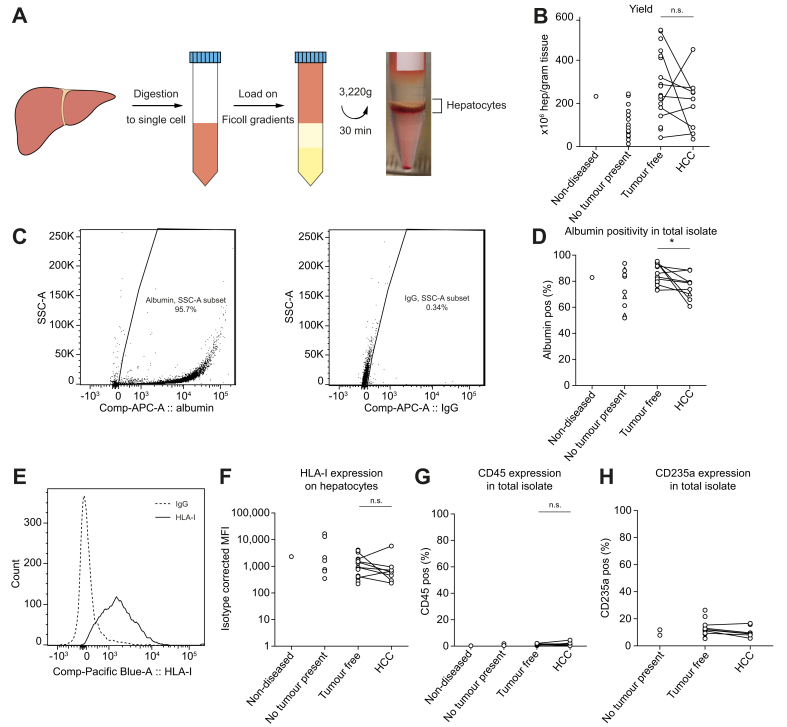
Hepatocyte isolation and characterisation. (A) Isolation procedure of primary hepatocytes. (B) Hepatocyte yields by trypan blue exclusion. ns: non-significant by the paired *t*-test. (C) Representative flow cytometric staining of albumin (left) with corresponding isotype (right). (D) Isotype-corrected albumin percentages in total isolates. Triangles represent samples stained with a discontinued antibody (Supplementary methods). ∗*p* <0.05 by the Wilcoxon matched-pairs signed-rank test. (E) HLA-I expression on albumin positive hepatocytes in a representative histogram and (F) across tissue types. (G) CD45 and (H) CD235a positivity in total isolates. F and G: n.s.: non-significant by the Wilcoxon matched-pairs signed-rank test. HCC, hepatocellular carcinoma. Comp-APC-A, allophycocyanin area; HLA-I, human leucocyte antigen class I; Pos, positive; MFI, mean fluorescence intensity; SSC-A, side scatter area.

To investigate the HLA-I peptidome of primary hepatocytes specifically, we developed a novel density centrifugation-based procedure to isolate hepatocytes from small clinical liver samples (Fig. 1A). This procedure yielded up to 540 × 10^6^ hepatocytes per gram of tissue (Fig. 1B) with an average of 213 ± 145 × 10^6^ hepatocytes (mean ± SD) across aetiologies including cirrhosis (Table 1).

**Table 1:** **Characteristics of hepatocyte donors**.

No.	Sex	anti-HBs	HBe	anti-HBe	Viral load (IU/ml)	HBV status	Antiviral	ALT (U/ml)	Fibrosis score	Differentiation grade HCC
**Non-diseased**										
1	ND	ND	ND	ND	ND	ND	ND	ND	NA	NA

**No tumour present**										
2	F	Neg	Neg	Pos	<2.0E1/N	C	ENT	70	No fibrosis	NA
3	M	14.3/P	Neg	ND	8.08E4/P	A	NA	2,206	Limited	NA
4∗	M	ND	Neg	Pos	1.18E3/P	C	NA	6,941	No fibrosis	NA
5	M	Neg	0.135/P	Pos	6.52E7/P	C	ENT	3,009	Fibrosis	NA
6	M	Neg	ND	ND	<2.0E1/N	C	TDF	22	Cirrhosis	NA

**Tumour-free tissue only**										
7∗	M	Neg	ND	Pos	<2.0E1/N	C	NA	33	Cirrhosis	Necrotic
8	M	Neg	Neg	Neg	<2.0E1/N	Neg	NA	ND	Cirrhosis	Moderate
9†	M	Neg	ND	ND	<2.0E1/N	Neg	NA	102	Cirrhosis	Moderate
10†	F	53.0/P	ND	ND	ND	R	NA	41	Cirrhosis	Moderate
11‡	M	Neg	ND	ND	<2.0E1/P	C	ENT	1,642	Cirrhosis	Necrotic
12‡	M	Neg	ND	ND	<2.0E1/P	C	TDF	14	Cirrhosis	Moderate
13	M	815/P	ND	ND	<2.0E1/N	R	TDF	60	Cirrhosis	NA
14	M	ND	Neg	Pos	<2.0E1/P	C	NA	27	Cirrhosis	NA
15	F	ND	0.330/P	Pos	<2.0E1/P	C	NA	14	No fibrosis	NA

**Tumour-free and paired HCC tissues**										
16	M	Neg	Neg	Pos	1.25E4	C	TDF	54	Cirrhosis	Poor
17	M	Neg	Neg	BD	<2.0E1/N	C	ENT	15	Moderate	Moderate
18	F	<3.0/N	ND	Neg	<2.0E1/N	R	NA	91	No fibrosis	Moderate
19‡	M	Neg	Neg	Pos	<2.0E1/P	C	TDF	24	No fibrosis	Moderate
20	M	ND	0.4030/P	Pos	5.87E4/P	C	TDF	51	Severe	Moderate
21	M	ND	ND	ND	ND	ND	NA	71	Limited	Moderate
22	M	Neg	Neg	Pos	1.12E5/P	AC	ENT	32	Cirrhosis	Moderate

/N, negative; /P positive; ALT, alanine aminotransferase; BD, borderline; ENT, entecavir; F, female; HCC, hepatocellular carcinoma; M, male; NA, not applicable; ND, not determined; TDF, tenofovir. Non-diseased: liver tissue rejected for transplantation owing to suspicion of steatosis. No tumour present: liver tissue of patients who did not have a liver tumour but underwent surgery for other medical reasons. Tumour-free tissue only: liver tissue adjacent to tumour lesions of which tumour tissue was not available for research purposes. Tumour-free and paired HCC tissues: both adjacent tumour-free and HCC tissue obtained from the same patient. HBV status: A: acute (positive [pos] for HBsAg and IgM anti-HBcAg); C: chronic (pos for HBsAg and anti-HBcAg); AC: acute in chronic background; R: resolved (HBsAg negative [neg] and anti-HBcAg pos).

∗ Donors 4 and 7 were positive for HDV RNA.

† Donors 9 and 10 were positive for IgG anti-HCV.

‡ Tumour-free tissues of donors 11, 12 and 19 are used in titration experiments.

Up to 95% of cells within isolates expressed albumin with an average of 80.6 ± 11.1% (Fig. 1C and D). Although albumin expression was lower in tumour isolates than in tumour-free isolates (*p* = 0.02), isolated albumin-positive hepatocytes expressed HLA-I to a similar degree across tumour and tumour-free samples (*p* = 0.38; Fig. 1E and F). Of note, HLA-II expression on isolated hepatocytes was assessed in a limited number of samples all in which expression was apparent (Supplementary materials & methods; Fig. S1). Importantly, contamination of CD45-expressing immune cells was negligible (1.1 ± 0.8%; Fig. 1G). As expected, because of their high density, the main contaminants in the isolates were CD235a^+^ erythrocytes (11.8 ± 5.1%; Fig. 1H). Because erythrocytes express extremely low HLA levels,^29^ further purification was not pursued.

### Identified peptides are HLA-I-derived and relate to liver function

**Fig. 2:**
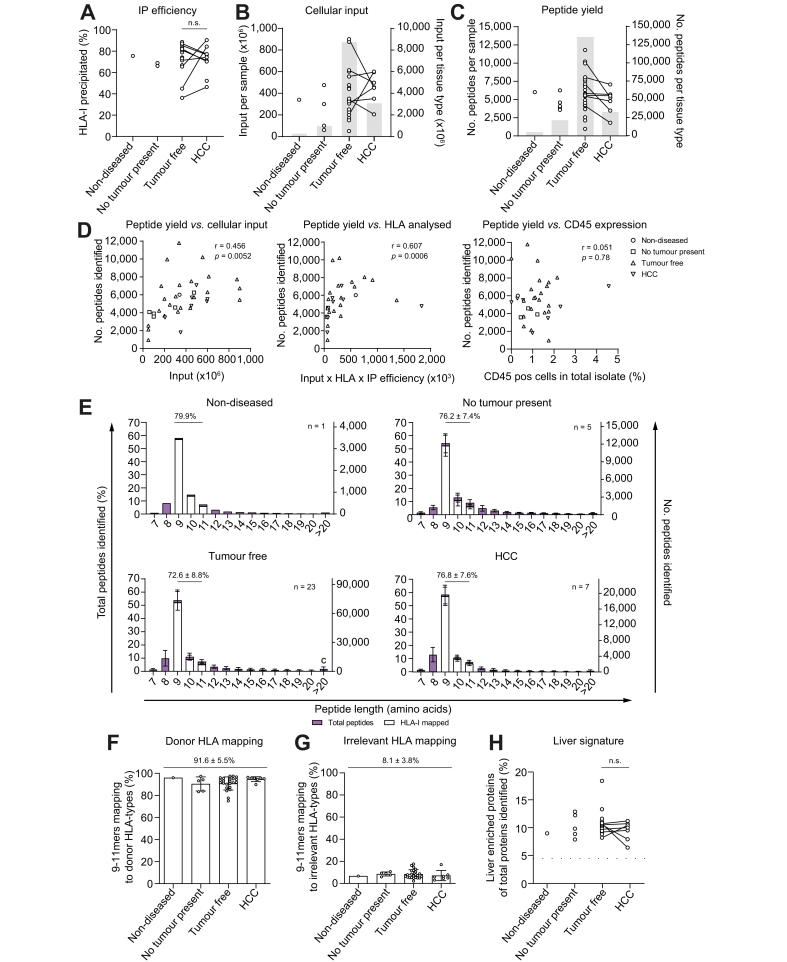
HLA-I immunoprecipitation monitoring. (A) IP efficiency by western blot. ns: non-significant by the Wilcoxon matched-pairs signed-rank test. (B) Hepatocyte input for IP per sample (individual data points; left y-axis) and cumulative across tissue types (grey bar diagrams; right y-axis). (C) Number of peptides identified per sample (individual data points; left y-axis) and cumulative across tissue types (grey bar diagrams; right y-axis). (D) Correlation of peptide yield with cellular input (left; Pearson correlation), cellular input corrected for HLA expression and IP efficiency (middle; Spearman correlation), or presence of CD45-expressing cells in the hepatocyte isolate (right; Spearman correlation). (E) The length distribution of identified peptides as percentage of total peptides identified (left y-axis) and as absolute number (right y-axis). The average percentage of 9–11mers ± SD is displayed above the graphs. 9–11mers mapping to donor HLA with a rank score ≤2.0% (NetMHCpan4.1) are distinguished in white. Error bars represent the SD. Percentage of 9–11mers (F) mapping to at least 1 donor HLA type or (G) mapping to HLA types for which the corresponding donor is negative. F and G: indicated is the overall mean ± SD. (H) Representation of liver-enriched proteins per tissue type as percentage of total source proteins presented in HLA-I. The percentage of liver-enriched proteins in the human genome (n = 936 out of 20,999) was 4.5% (dotted line). n.s.: non-significant by the Wilcoxon matched-pairs signed-rank test. HCC, hepatocellular carcinoma; HLA, human leucocyte antigen; HLA-I, human leucocyte antigen class I; IP, immunoprecipitation.

Next, we isolated HLA-I complexes using immunoprecipitation (IP) with an average efficiency of 72.2 ± 13.1% (mean ± SD; Fig. 2A; Supplementary materials & methods; Fig. S2A). Titrations indicated that DDA peptide yield was greatly favoured by high cellular input and was not easily saturated (Supplementary materials & methods; Fig. 2B). We therefore used all hepatocytes available for IP (Fig. 2B). Of all peptides identified by a database search, 1.6% (n = 3,270) were derived from *S. aureus*. The majority of these (>98.0%) unambiguously mapped (*i.e.* unique for a single protein-coding gene) to the Protein A used in the IP procedure. All *S. aureus* peptides were therefore excluded from further analysis. In addition, peptides without an accession annotation (n = 1,816; 0.9% of total peptides identified) or with a murine annotation (n = 2) were excluded. Analysis was continued with the remaining 198,701 peptide sequences that were identified in a total of 36 samples with an average of 5,520 peptides per sample (Fig. 2C).

Peptide yield and cellular input were significantly correlated (Fig. 2D, left panel; r = 0.456; *p* = 0.005) and correlated even stronger when input was corrected for HLA-I expression and IP efficiency (Fig. 2D, middle panel; r = 0.607; *p* = 0.0006). Importantly, peptide yield did not correlate with the remaining presence of CD45-expressing immune cells in the isolate (Fig. 2D, right panel; r = 0.051; *p* = 0.78), further indicating that identified peptides originated from hepatocytes. Analysis of peptide length frequency revealed a length distribution of primarily 9–11mers, which is typical for HLA-I peptides (Fig. 2E). On average, 91.6 ± 5.5% of all 9–11mers were predicted using the NetMHC prediction algorithm to contain an HLA-binding motif for at least 1 HLA type the donor was positive for (Fig. 2F; Supplementary materials & methods; Table S1). In contrast, only 8.1 ± 3.8% of identified 9–11mers contained a binding motif for an irrelevant HLA type (Fig. 2G; Supplementary materials & methods; Table S1). Importantly, liver-related proteins (from Human Protein Atlas) were significantly enriched in HLA peptidomes when compared with their presence in the human genome (Fig. 2H; *p* <0.0001 for each tissue type), and enrichment did not differ significantly between tumour and tumour-free tissues (*p* = 0.89). Together, these data strongly indicate that identified peptides are predominantly *bona fide* hepatocyte-derived HLA peptides.

Next, Gene Ontology (GO) analysis and Kyoto Encyclopedia of Genes and Genomes (KEGG) pathway analysis were performed to gain more insight into the function and cellular localisation of source proteins. In total, 220 GO terms were significantly enriched across HLA peptidomes compared with the human genome. The most relevant GO terms pointed towards prevalent presentation of proteins involved in (viral) transcription, adhesion, and HLA-I antigen presentation from cellular locations such as the nucleus, mitochondrion, cytoplasm, and ribosomes (Supplementary materials & methods; Fig. S3). Consistent with the essential functions of hepatocytes, KEGG pathways highlighted abundant presentation of proteins involved in metabolism, protein translation, and blood coagulation (Fig. S4). Notably, pathways related to cancer and viral infection were additionally enriched.

### Presentation of HBV-derived peptides

Liver tissues of 12 out of 19 donors with (a clinical history of) HBV infection or unknown HBV status were HBsAg positive (Supplementary materials & methods; Fig. S5; Tables S2 and S3), yet only 8 HLA-I peptides were HBV-derived. These peptides originated from viral proteins HBsAg and DNA polymerase (Pol) and were detected in tumour-free and HCC isolates from 4 patients (Table 2; Supplementary materials & methods; Fig. S6). All tissues in which HBV-derived peptides were detected were HBsAg positive except for that of donor 18 who resolved HBV infection. HBV-derived peptides were examined in detail to identify potential therapeutic targets. Importantly, the well-described epitopes HBsAg_183-191_ and HBsAg_313-321_ were detected in the eluate of the HBsAg-positive HCC tissue from a patient with acute-on-chronic HBV infection (Table 2; Supplementary materials & methods; Table S3; Fig. S5). Both of these epitopes were predicted to bind the patients’ HLA type. However, the predicted HLA origin of HBsAg_183-191_ did not match the HLA context it was previously reported for (*i.e.* HLA-A∗02 supertype). Instead, HBsAg_183-191_ was likely presented by this patients’ B or C alleles (Table 2). In addition, 6 HBV peptides were identified that were not yet reported in the IEDB.^9^

**Table 2:** **Peptides identified from HBV-derived antigens**.

				Donor samples∗					
Antigen	Position	Sequence†	HLA type‡	TF	HCC	HBV§	Viral load (IU/ml)	IEDB¶	PRM∗∗
HBsAg	6–17	STSNPLGFFPDH	NB	20		C	5.87E4/P		N
HBsAg	14–23H	FPDHQLHPAF	B∗08:01; B∗35:02; C∗04:01		22	AC	1.12E5/P		VG
HBsAg	14–23D	FPDHQLDPAF	B∗35:02; C∗04:01		22	AC	1.12E5/P		VG (2)
HBsAg	**183–191**	**FLLTRILTI**	**B∗08:01;** **C∗04:01;** **C∗07:01**		**22**	**AC**	**1.12E5/P**	**◊**	**VG (3)**
HBsAg	**313–321**	**IPIPSSWAF**	**B∗08:01** **B∗35:02;** **C∗04:01**		**22**	**AC**	**1.12E5/P**	**○●**	**N/A**
HBsAg	256–264	RGPNLYSTL	A∗24:02 C∗07:02 C∗15:05	7		C	<2.0E1/N		N/A
Pol	11–21	LLLLDDDAGPV	A∗02:01		18	R	<2.0E1/N		N/A
Pol	705–714	RGTFVSPLPI	B∗57:01		18	R	<2.0E1/N		N/A

DDA, data-dependent acquisition; IEDB, Immune Epitope Database; IFNγ, interferon γ; Pol, polymerase; PRM, parallel reaction monitoring; TF, tumour-free.

∗ Donor tissue in which the corresponding peptide is identified.

† Underscored amino acids are not in consensus with the NC_003977.2 reference sequence.

‡ NB: not predicted to bind donor HLA.

§ HBV status of patients in which peptides are identified: AC: acute-on-chronic infection; C: chronic infection; R: resolved infection.

¶ Whether a sequence is reported as HLA ligand in the IEDB or not. For reported sequences, it is indicated whether they are identified in a new context of HLA supertypes they were not previously associated with (◊) or in the context of both the described and another HLA supertype (○●). Peptides in bold are listed as immunogenic in the IEDB (*i.e.* positive multimer binding and/or IFNγ production).

∗∗ VG: Peptides with a *very good* match in the same sample as the DDA identification. The number of very good matches in additional samples is indicated between brackets (Supplementary Materials & methods; Table S5). N: not detected with PRM. N/A: peptides not included in the target list for PRM analyses.

The unbiased DDA approach was followed up with targeted PRM to screen all samples for the identified HBV-derived peptides with a higher sensitivity. For this purpose, only peptide–spectrum matches of high quality were included. Four out of 8 HBV-derived peptides were associated with MS fragmentation spectra of sufficiently high quality to include in the selection for downstream PRM analyses (Supplementary materials & methods; Fig. S6). PRM results were manually ranked into 5 confidence categories based on the number of fragment ions, their relative intensities, and their corresponding elution profiles (Supplementary materials & methods and Fig S7), of which only confidence categories 4 (good match; Table S4) and 5 (very good match; Table 2; Supplementary materials & methods; Table S4) were considered further. Of the 4 selected HBV-derived peptides, 3 were identified using PRM. As expected, these identifications were exclusively made in HBV-infected tissues. Furthermore, all 3 peptides were identified at the highest confidence category in the sample they had been originally identified in by DDA (Table 2; Supplementary materials & methods; Table S4). In addition, HBsAg_14-23D_ and HBsAg_183-191_ were identified with category 5 confidence in 2 and 3 additional samples, respectively (Table 2; Supplementary materials & methods; Table S4). Importantly, these identified peptides matched at least 1 of the patients’ HLA type in 4 out of 5 cases (Supplementary materials & methods; Table S4) and extended our DDA results with the finding that both HBsAg_14-23D_ and HBsAg_183-191_ are also presented in non-tumourous HBV-infected liver tissues. In addition, 1 lower confidence identification was made for HBsAg_183-191_ (Supplementary materials & methods; Table S4).

### Presentation of tumour-specific antigens

**Fig. 3:**
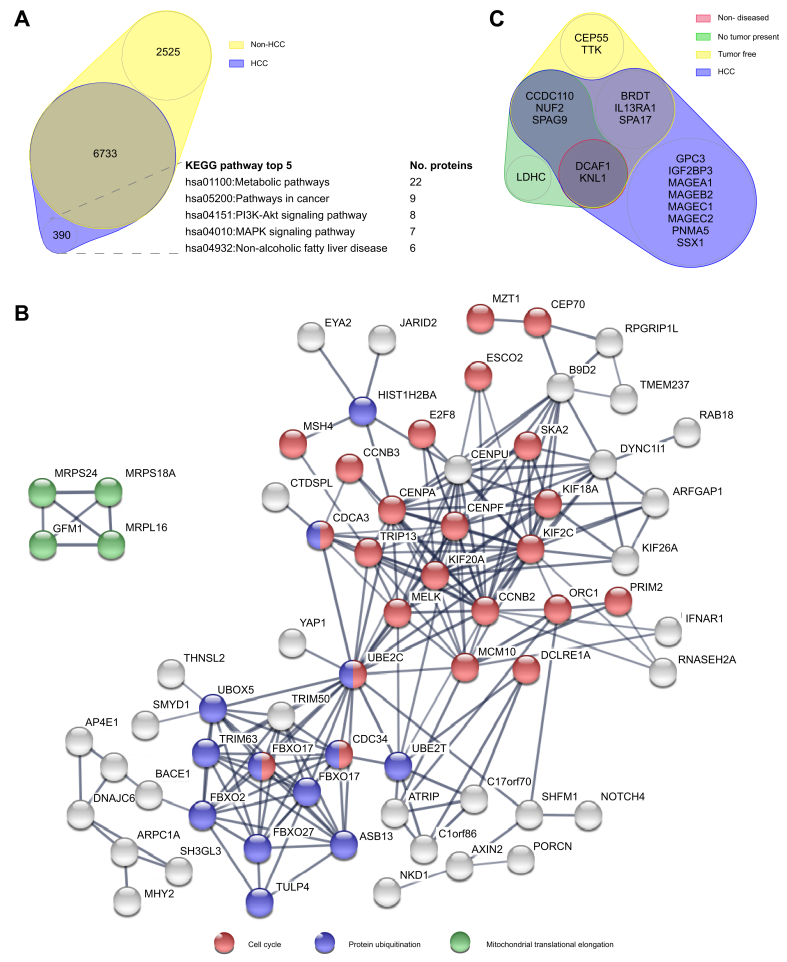
Identification of tumour-associated antigens. **(**A) Euler diagram. Circle sizes represent the absolute number of source proteins identified in all non-HCC and HCC tissues. Best covered KEGG pathways by HCC-exclusive proteins are indicated. (B) STRING protein–protein interaction network of HCC-exclusive source proteins with at least 3 protein interactions and their connecting nodes. The width of an edge connecting 2 nodes represents the interaction confidence (≥0.7). Nodes annotated to 3 main GO molecular functions are color-coded as indicated. (C) Euler diagram. Circle sizes represent the number of described HCC-associated tumour-associated antigens in each tissue. GO, Gene Ontology; HCC, hepatocellular carcinoma; KEGG, Kyoto Encyclopedia of Genes and Genomes.

Next, we focussed on the 390 proteins that were exclusively presented by HCC-derived hepatocytes (Fig. 3A). Although KEGG pathway analysis did not reveal significantly enriched pathways, the top 5 pathways covered by these proteins involved metabolism as well as oncogenesis. STRING analysis of protein–protein interactions further revealed 2 highly connected clusters of HCC-restricted proteins that were engaged in ‘cell cycle’ and ‘protein ubiquitination’ (Fig. 3B). These clusters were connected via the E2 ubiquitin-conjugating enzyme UBE2C, which was the most connected protein of the network with 22 direct interactors. In addition, a smaller cluster involved in ‘mitochondrial protein elongation’ was identified. Other protein clusters did not clearly map to a particular GO biological process.

We then explored presentation of peptides derived from described TAA by hepatocytes isolated from different tissue types (Fig. 3C). A previous systematic literature search yielded 107 TAA with known HCC association,^30^ from which all identified peptides were extracted from the immunopeptidome. A total of 129 peptides from 19 HCC-associated TAA were identified. Peptides from 11 source proteins were detected in HLA eluates from non-tumour tissues, thereby discarding these 11 TAA as suitable therapy targets for HCC (Supplementary materials & methods; Table S5).

Importantly, 14 peptides from 8 TAA were unambiguously identified exclusively in HLA eluates from HCC tissues (Fig. 3C; Table 3). Although none of these peptides were associated with HCC before, 7 were described in other tumour types. Only 2 identified TAA-derived peptides were listed in the IEDB as immunogenic (Table 3, bold); 7 unique peptides from known TAA were completely novel.

**Table 3:** **Peptides derived from known tumour-associated antigens exclusively identified in HLA-I eluates from HCC tissues by unbiased MS**.

Antigen	Position	Sequence	HLA type∗	Donor	HLA†	Tumour‡	PRM§
GPC3	376-384	AHVEHEETL	B∗38:02 C∗07:01 C∗07:02	16			VG
GPC3	522-541	FLAELAYDLDV-DDAPGNSQQ	NB	21			N/A
GPC3	531-541	DVDDAPGNSQQ	NB	21			VG
IGF2BP3	552-560	KIQEILTQV	A∗02:01	17	○	Brain; breast; Mel; ML; ovarian	VG (1 HCC)
MAGEA1	**278-286**	**KVLEYVIKV**	**A∗02:01** **C∗07:01**	**17**	**○●**	**Mel; uterine**	**VG (1 HCC; 1 TF)**
MAGEA1	301-309	ALREEEEGV	A∗02:01	17	○	Mel	VG
MAGEB2	**231-240**	**GVYDGEEHSV**	**A∗02:01** **C∗04:01**	**18**	**○●**	**Mel; ML; cervix; uterine**	**VG**
MAGEC1	611-621	FPQSPLQGEEF	B∗35:02 C∗04:01	22			VG
MAGEC1	1,035-1,043	FAFGEPREL	A∗68:02 B∗52:01 B∗78:01 C∗16:01	19	○●	Brain; Mel	VG
MAGEC1	1,061-1,069	NSSPPRYEF	A∗68:02 B∗52:01 B∗78:01 C∗16:01	19	Δ	Mel	VG
MAGEC2	176-184	DYFPVILKR	A∗33:01	19			VG
PNMA5	61-69	NAKAVFIEL	A∗68:02 B∗52:01 B∗78:01 C∗16:01	19	**●**	Breast	G
SSX1	23-32	KAFDDIATYF	B∗57:01 C∗04:01 C∗07:04	18			VG
SSX1	42-50	YSEKISYVY	A∗01:01 B∗35:02 C∗04:01 C∗07:01	22			N/A

DDA, data-dependent acquisition; HCC, hepatocellular carcinoma; IEDB, Immune Epitope Database; IFNγ, interferon γ; MS, mass spectrometry; PRM, parallel reaction monitoring; TF, tumour-free.

∗ NB: not predicted to bind donor HLA.

† Whether a sequence is reported as HLA ligand and/or epitope in the IEDB or not. For reported sequences, it is indicated whether they are identified exclusively in a new (●), the same (○), or in addition to another (○●) HLA-supertype context as previously reported. Some sequences are reported in the IEDB but lack a specified HLA-type entry (Δ). Peptides in bold are listed as immunogenic in the IEDB (*i.e.* positive multimer binding and/or IFNγ production).

‡ Tumour types for which this peptide was reported as HLA ligand and/or epitope in the IEDB. Mel: melanoma; ML: myeloid leukaemia.

§ VG: Peptides with a 'very good match' in the same sample as the DDA hit. The number of very good matches in additional samples is indicated between brackets also stating the tissue type (see Supplementary Materials & methods; Table S7). G: peptide with a good match in the same sample as the DDA hit. N/A: peptides not included in the target list for PRM analyses.

We again followed up on our unbiased MS approach with PRM to identify thus far HCC-specific 9–11mers in all samples across our patient cohort. The spectrum of SSX1_42-50_ was of insufficient quality for reliable PRM analysis and was therefore excluded from PRM data acquisition (Supplementary materials & methods; Fig. S8). Of the 12 peptides included in the target list for PRM, 11 were confirmed in the sample of origin with high confidence category 5 (Table 3; very good match). In addition, high confidence identifications were obtained for IGF2BP3_552-560_ and MAGEA1_278-286_ in 1 and 2 other samples, respectively (Table 3; Supplementary materials & methods; Table S6). One of the latter was made in a patient with cHBV but without HCC. Of note, we also identified IGFBP3_552-560_ in tissue of a patient not having HCC at all (Supplementary materials & methods; Table S6), albeit with less confidence (category 4; good match).

### Immunogenicity of HBV- and TAA-derived peptides

**Fig. 4:**
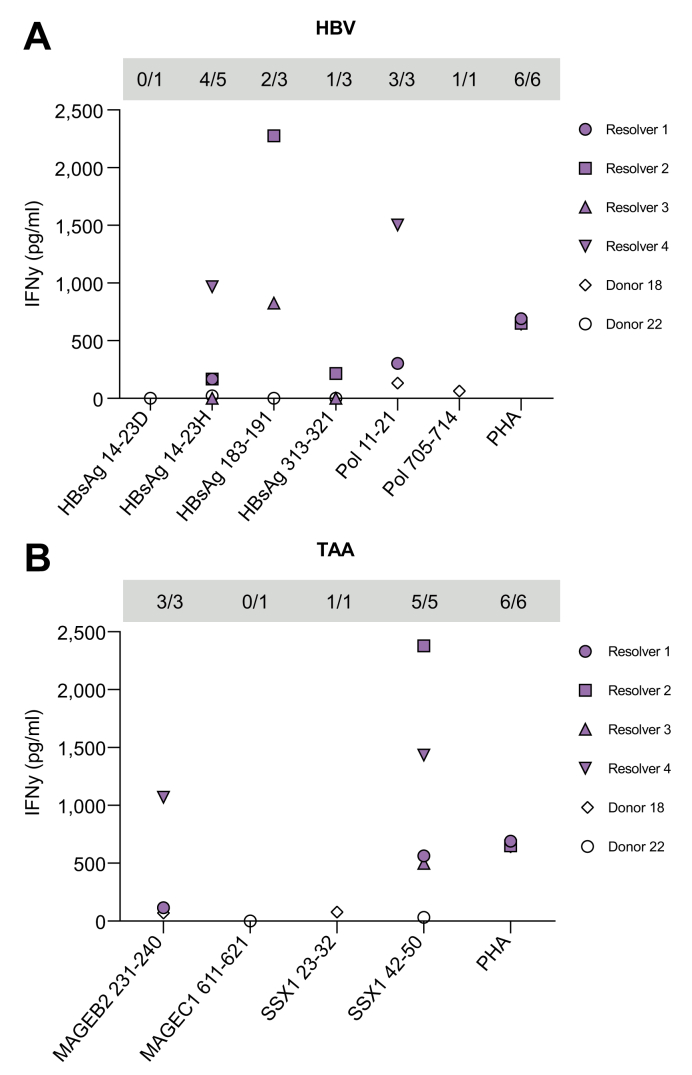
Immunogenicity of HLA-presented peptides. Expanded PBMCs from 4 HBV resolvers and 2 patients with HBV/HCC were restimulated with peptides derived from (A) HBV or tumour (B) antigens. IFNγ concentrations were measured using ELISA and corrected for background (vehicle). Grey boxes present the number of responsive donors (background corrected mean ≥ mean + 3 × SD of vehicle) *vs*. subjects tested. HCC, hepatocellular carcinoma; HLA, human leucocyte antigen; IFNγ, interferon γ PBMC, peripheral blood mononuclear cell; PHA, phytohemagglutinin; Pol, polymerase; TAA, tumour-associated antigens.

To further investigate the therapeutic potential of identified HBV-derived peptides, we evaluated IFNγ production by PBMCs after peptide-specific expansion for the 2 donors in which HBV-derived peptides were detected and PBMCs were available (*i.e.* donors 18 and 22 in which 2 and 4 different peptides were identified, respectively; Table 2). In addition, 4 HLA-matched individuals (Supplementary materials & methods; Table S7) who had previously cleared HBV infection (positive for anti-HBsAg and anti-HBcAg) were included to expand the dataset. Peptide-specific IFNγ production was found in response to 5 out of 6 HBV peptides tested (*i.e.* >3× SD above background; Fig. 4). In general, peptide-induced IFNγ production was lower in patients than in HBV resolvers, despite similar overall T cell fitness (Supplementary materials & methods; Fig. S9). Yet, 3 completely novel HBV epitopes were identified: HBsAg_14-23H_, Pol_11-21_, and Pol_705-714_.

Moreover, 2 HBV resolvers were highly responsive to the well-known HLA-A∗02 epitope HBsAg_183-191_ (Fig. 4A). Like patient 22 in which this peptide was identified by MS, these resolvers were negative for HLA-A∗02 but positive for HLA-B∗08:01 and HLA-C∗07:01, from either which this peptide is likely to originate based on *in silico* prediction (Table 2). This finding demonstrates immunogenicity of HBsAg_183-191_ for the first time in a non-HLA-A∗02 context.

Similarly, we evaluated the immunogenicity of TAA-derived peptides presented on hepatocytes in these same 2 patients (2 different TAA peptides in each donor; Table 3). IFNγ production was observed in response to 3 out of 4 TAA-derived epitopes: MAGEB2_231-240_, SSX1_23-32_, and SSX1_42-50_ (Fig. 4B). This is a first demonstration of immunogenicity for these SSX1 epitopes and shows the immunogenicity of MAGEB2_231-240_ for the first time in a patient with HBV/HCC. Because responses against MAGEB2_231-240_ and SSX1_42-50_ were identified in several donors, it is likely that these responses depended on HLA types commonly expressed by all donors (*i.e.* HLA-A∗02:01 for MAGEB2_231-240_ and HLA-A∗01:01 and/or HLA-C∗07:01 for SSX1_42-50_).

## Discussion

To study HLA-I presentation by primary hepatocytes, we have developed a novel procedure to isolate primary human hepatocytes without perfusion. Our novel isolation procedure yielded an averaged 15 times more hepatocytes per gram of tissue compared with optimised perfusion (13 × 10^6^ cells/g).^31^ This yield allowed for the first large-scale analysis of the HLA-I peptidome of isolated, immune cell-depleted, patient-derived hepatocytes and tumour cells. Our workflow delivers a valuable resource for the development of generic or personalised forms of antigen-specific immunotherapies for liver diseases in general.

The current study yielded an extensive, high-quality peptidome with percentages of 9–11mers and HLA-binding properties similar to previous reports.^3^^,^^17^^,^^32^ In line with other studies, we detected overrepresentation of source proteins with high turnover (*e.g.* involved in the Golgi apparatus) and of specific cellular localisation (nucleus, mitochondria, and ribosomes).^32^^,^^33^ Percentages of contaminating immune cells were minimal and did not correlate with peptide yield, rendering it highly unlikely that immune cells contributed significantly to obtained peptidomes. Instead, peptide yield correlated with hepatocyte input and HLA expression levels. Moreover, source proteins were largely involved in hepatocyte-related processes, and many were previously denoted as liver-related.^25^ Taken together, these data strongly indicate that identified peptides were derived from hepatocyte HLA-I complexes.

Some studies have already reported on HBV-derived peptides in immunopeptidomes of HBV-related HCC or cell lines that artificially express HBV proteins.^7^^,^^34^ Dong *et al*.^7^ investigated the HLA peptidome of the entire HCC tissue and identified dozens of HBV peptides from each patient. However, the total HLA peptide yield and the predicted ability of peptides to bind donor HLA were considerably lower compared with our study (20,927 *vs*. 198,701 total peptides and 63 *vs*. 92% of HLA mapping). Moreover, samples analysed by Dong *et al.*^7^ contained tumour-infiltrating and/or liver-resident leucocytes, which have far superior HLA-I expression compared with hepatocytes. Therefore, HBV-derived peptides might have originated from immune cells instead of hepatocytes. This notion is further supported by our finding that HLA peptide yield from dendritic cells is generally 10 times higher than that of hepatocytes (data not shown). Confounding immune cell presence may even have been augmented by local ablative pretreatment of some patients before surgery, which attracts immune cells.^35^ Although the peptidome described by Dong *et al.*^7^ provides an elegant analysis of HLA-I presentation in whole tissue, the peptidome described here is far more representative of hepatocyte-specific antigen presentation, which is a crucial factor in the development of effective antigen-specific immunotherapy.

Despite our relatively low HBV-derived peptide yield, we were still able to identify 8 HBV-derived peptides in immune depleted hepatocyte isolates. Our data now provide compelling evidence that these peptides are presented on infected and/or malignant target cells and that at least 3 of the 6 completely novel HBV peptides are immunogenic. However, immune responses in patients with HBV/HCC were considerably lower than those in HBV resolvers despite similar overall T cell fitness. This possibly points to HBV antigen/TAA-specific T cell dysfunction, deletion, and/or inefficient T cell priming in these patients.^36^ Unfortunately, we did not have sufficient material to assess this in more detail.

Identified HBV peptides originated exclusively from HBsAg and Pol and not from HBeAg, HBcAg, or HBx. This is not unexpected, as (1) HBx is short, truncated in HCC and expressed at low levels,^37^ and (2) HBeAg/HBcAg expression may have been low, as 3 out of 4 patients in which HBV peptides were identified were HBcAg negative in the liver, serum HBeAg negative, and/or anti-HBeAg positive. Among HBsAg peptides, we identified well-described epitopes HBsAg_183-191_ and HBsAg_313-321_ for the first time in HLA eluates from primary liver cells. HLA-A∗02–HBsAg_183-191_ complexes were previously detected in liver biopsies of patients with cHBV and could drive regression of HBV-related HCC lesions when targeted by adoptively transferred T cells.^14^^,^^38^^,^^39^ In the present study, HBsAg_183-191_ was predicted to originate from HLA-B∗08 and HLA-C types. Concordantly, we showed immunogenicity in HLA-A∗02 negative but HLA-B∗08:01 and HLA-C∗07:01 positive donors. Our experimental setup and the donors at our disposal to assess immunogenicity did not allow distinction among these HLA types or exclude involvement of HLA-II/CD4^+^ T cells. However, HLA-B∗08:01 is our prime candidate for the presentation of HBsAg_183-191_ on hepatocytes and IFNγ production in the *in vitro* assay for several reasons: HLA-A∗02 and HLA-B∗08 share binding properties,^40^ HLA-C alleles contribute less to the HLA peptidome,^18^ and HLA-II preferably binds longer peptides. Targeting HBsAg_183-191_ might thus also be beneficial in context of HLA-B∗08:01 and/or HLA-C∗07:01.

In addition, 2 HLA peptides originating from Pol were identified in the tumour HLA eluate of an HLA-A∗02:01-positive patient who presumably resolved infection (*i.e.* apparent absence of HBV protein expression; cleared HBsAg and viral DNA). Identification of HBV peptides in this presumed resolver could still point to a lingering subclinical infection, matching the lack of HBsAg- and HBeAg-directed antibodies in this patient. Alternatively, identified Pol-derived peptides may have been expressed from integrated DNA.^14^^,^^39^ HLA peptides from integrated DNA are of high interest for immunotherapy of HCC as these could be specific tumour targets independent of active infection.

Previous HLA peptidome studies in HCC primarily focused on identification of neo-epitopes as immunotherapeutic targets for personalised treatment.^3^^,^^7^ However, the study of neo-epitopes is challenging because HCC has a low mutational burden^5^ and infrequent HLA-I neo-epitope presentation.^3^ In addition, immune cell presence in whole tissue may further complicate the detection of neo-epitopes presented by hepatocytes.^3^^,^^20^ Here, we present a novel workflow that may resolve this issue, as it yields large quantities of isolated primary hepatocytes from small-scale surgical resection materials. Our study can herewith additionally contribute to the development of personalised immunotherapy directed at neo-antigens, especially in an adjuvant setting.

Tumour material, time, and resources for personalised therapy development will, however, not always be available, and classical, non-mutated TAA may allow for a more generic approach. Recently, interest in such antigens was revived by a promising clinical study in melanoma targeting TAA with an mRNA vaccine.^41^ In the present study, HCC-exclusive peptides from 8 described TAA were identified despite relative undersampling with respect to tumour-free tissues. PRM analysis for peptides from IGFBP3 and MAGEA1 showed probable or definite presence in material from patients without a tumour. These findings suggest that expression of IGFBP3 and MAGEA1 may not be HCC-specific after all or that these tissues may be premalignant.^30^ Of the remaining HCC-specific peptides, we were able to test a selection of 4 TAA-derived peptides for immunogenicity. Albeit of low magnitude, immune responses against 3 TAA-derived peptides were detected in the patients with HBV/HCC in whose HLA eluates these peptides were identified. Additionally, TAA responses were detected in HBV resolvers. It is unclear whether these responses relate to HBV resolver status, but finding TAA responses in non-cancerous individuals is not uncommon.^42^ Whether such responses can protect against tumour formation is unknown. Still, the identified HBV- and TAA-derived epitopes are highly promising targets for multiple types of immunotherapy such as adoptive T cell transfer or vaccination strategies.^43^^,^^44^

Several of the HCC-exclusive TAA-derived peptides have demonstrated relevance for other cancers but had not yet been associated with HCC. Conversely, HLA peptides from several reported TAA were also detected in non-tumour tissues, thereby revoking their use as immunotherapeutic targets. Our data thus contribute directly to expansion and selection of the TAA HLA peptide repertoire for the development of effective generic antigen-based anti-tumour immunotherapy.

Precedented by our current demonstration that HLA peptidome analysis from isolated hepatocytes is feasible, the advance of quantitative and targeted MS approaches may soon put a comparison of hepatocyte *vs*. immune cell presentation within reach.^45^ In this equation, HLA-II presentation is also an important factor.[46], [47], [48] In the liver specifically, non-parenchymal cells such as liver sinusoidal cells (LSECs), Kupffer cells, and dendritic cells play an important role in this process because they can modulate both CD4^+^ and CD8^+^ T cell responses.^49^ However, antigens may not be presented in the right context for immunogenic T cell priming.^50^ It would therefore be interesting to compare the HLA-I and HLA-II immunopeptidomes of different antigen-presenting cell subsets that reside in the liver to determine in which immunomodulatory context specific antigens are presented. Answering this pivotal question may help explain why T cells of certain specificities in cHBV are of poorer quality than others.[51], [52], [53]

Taken together, our study extensively characterised HLA-I peptides presented by isolated hepatocytes from HBV-infected livers and HCC in relation to benign samples. Our data rationally highlight a variety of HBV- and TAA-derived HLA-I peptides to aid the future development of effective antigen-specific immunotherapies against cHBV and (HBV-related) HCC.

## Financial support

This work was financed by a unique high-risk grant from the 10.13039/501100004622Dutch Cancer Society (10429) and the Dutch Ministry of Economic Affairs and Climate Policy by means of the public private partnership (PPP) allowance from the Top Sector Life Sciences & Health to stimulate public–private partnerships in conjunction with the Dutch Digestive Foundation (LSHM16056). In the latter, ISA Pharmaceuticals B.V. Leiden, the Netherlands, is the collaborating and co-funding private partner.

## Authors’ contributions

Methodology: MTAdB, KB, RB, JAAD, SIB. Formal analysis: MTAdB, KB, RL, WASD, PPCB, RFAP, JAAD. Investigation: MTAdB, KB, RL, PPCB, RFAP, PJB, MD. Data curation: MTAdB, WASD, JAAD. Validation: MTAdB, JAAD, SIB. Writing – original draft: MTAdB. Writing – review and editing: MTAdB, JNMIJ, MD, RAdM, AMW, JAAD, SIB. Visualisation: MTAdB. Resources: JNMIJ, MD, SIB. Supervision: RAdM, AMW, SIB. Funding acquisition: AMW, SIB. Conceptualisation: SIB.

## Data availability statement

The mass spectrometry proteomics data obtained by DDA have been deposited to the ProteomeXchange Consortium via the PRIDE^28^ partner repository with identifier PXD023143 and is publicly available at http://www.ebi.ac.uk/pride.

## Conflicts of interest

SIB collaborates with and receives co-funding from ISA Pharmaceuticals B.V., Leiden, the Netherlands. SIB and MTAdB are listed as inventors on a patent application filled by ISA Pharmaceuticals B.V. The authors declare no further conflicts of interest.

Please refer to the accompanying ICMJE disclosure forms for further details.
