# Supplementary material for: Immunopeptidome of hepatocytes isolated from patients with HBV infection and hepatocellular carcinoma
Source: JHEP Rep. 2022 Sep 5;4(11):100576. doi: 10.1016/j.jhepr.2022.100576 (PMC9523389; doi:10.1016/j.jhepr.2022.100576)
Supplement: Multimedia component 1 [file mmc1.pdf]

Immunopeptidome of hepatocytes isolated from patients with HBV infection and hepatocellular carcinoma

Monique T.A. de Beijer, Karel Bezstarosti, Robbie Luijten, Wouter A.S. Doff, Patrick P.C. Boor, Roel F.A. Pieterman, Rachid Bouzid, Paula J. Biesta, Jan N.M. Ijzermans, Michail Doukas, Robert A. de Man, Andrea M. Woltman, Jeroen A.A. Demmers,

Sonja I. Buschow

Table of contents

Patients and methods 4

Patient material 4

Isolation of patient-derived hepatocytes 5

Phenotyping of hepatocyte- containing isolates 5

Conjugation of pan HLA-I antibody to Protein A Sepharose beads 6

Purification of HLA-I complexes 6

Immunoprecipitation efficiency by western blot 7

Sample preparation and LC-MS/MS data acquisition 7

Bioinformatics analysis of DDA data 9

Gene ontology, KEGG pathway and STRING analysis 9

Bioinformatics analysis of PRM data 10

Immunohistochemistry 11

Immunogenicity 11

T cell fitness 12

HLA typing 13

Supplementary figures 14

Fig. S1 14

Fig. S2 15

Fig. S3. 16

Fig. S4. 17

Fig. S5. 18

Fig. S6. 19

Fig. S7. 20

Fig. S8. 22

Fig. S9. 23

Supplementary tables 24

Table S1 24

Table S2 25

Table S3 25

Table S4 26

Table S5 27

Table S6 29

Table S7 29

Supplementary references 30

PATIENTS AND METHODS

Please refer to the CTAT method tables for a detailed overview on antibodies, patient materials and software used.

Patient material – Samples of human liver tissue were obtained from 21 patients undergoing liver transplantation or surgical resection at the Erasmus Medical Center in Rotterdam, the Netherlands. Use of this material for our studies was approved by the local ethics committee (MEC2014-060 for explants from liver transplantation / MEC2009-012 for resection material). Liver material was obtained from patients who did not have a tumor but underwent surgery for other medical reasons (no tumor present; n=5) and from patients with liver tumors from whom tumor material was available for research purposes (tumor free and paired HCC tissues; n=7) or not (tumor free only; n=9). Some tumor free tissues were used in titration experiments which accounts for another 7 samples. Patient samples were included based on availability of personnel and tissues as well as tissue quality (i.e. non-necrotic). Distinction between tumor and tumor free tissue was made by an experienced pathologist. Tumor free tissue was obtained at least 2 cm from the tumor nodule. In addition, one sample was derived from a non-HCC/HBV affected liver that had been rejected for transplantation because of suspicion of steatosis as assessed by an experienced transplant surgeon (non-diseased; n=1). Table 1 provides more detailed characteristics of all donors.

Isolation of patient-derived hepatocytes – Liver samples were cut into small pieces and digested for 40 min shaking at 37°C in Williams E Medium without glutamine (Gibco) supplemented with 8% fetal calf serum (FCS; Sigma), 1% penicillin/streptomycin (Gibco), 1% MEM non-essential amino acids solution 100x (MEM-NEAA, Gibco), 1% sodium pyruvate (Gibco), 0.5 mg/ml Collagenase from Clostridium histolyticum type IV (Sigma) and 0.2 mg/ml DNAse I from bovine pancreas (Roche). Samples were processed through a 100 µm filter in presence of 0.5 mM EDTA (Lonza) and cOmplete™ Mini EDTA-free Protease Inhibitor Cocktail tablets (Roche) according to the manufacturer's instructions. Cell suspensions were loaded on a 1.077 g/ml Ficoll Paque Plus gradient routinely used for leukocyte isolation (GE Healthcare) stacked upon 1.2 g/ml Ficoll PM400 (GE Healthcare) in PBS (Lonza). Hepatocytes were isolated by harvesting the interphase after a 30 min spin at 4°C at 3220 g and stored as dry pellets at -80°C until HLA-I purification.

Phenotyping of hepatocyte- containing isolates – Isolates were blocked in 50 µg/ml human Fc block (BD) for 10 min in the dark at room temperature (RT) and subjected to phenotypic staining for 30 min at 4°C in PBS + 1% FCS + 2 mM EDTA + 0.02% sodium azide (Sigma) using a R-phycoerythrin (R-PE)- conjugated antibody to CD235a (JC159), a pacific blue (PB)- conjugated antibody to HLA-I (w6/32) or corresponding isotype (MOPC-173), a Brilliant Violet (BV421)- conjugated antibody to HLA-II (Tu39) or corresponding isotype (G155-178) and allophycocyanin (APC)-, allophycocyanin – eFluor780 (APC-eFluor780)-, or phycoerythrin (PE)- conjugated antibodies to CD45 (HI30, HI30 and J33 respectively). Intracellular staining was performed after fixation for 15 min at RT in 4% paraformaldehyde (PFA; Sigma) and permeabilization for 15 min at RT in 0.5% saponin (Sigma). Samples of the first 6 liver samples were subsequently

blocked for 15 min at RT in 10% donkey serum (Abcam) and stained using a polyclonal goat anti-albumin antibody or corresponding isotype for 30 min at RT followed by a polyclonal AF647-conjugated donkey anti-goat antibody for 30 min at RT. Due to discontinuation of the primary antibody, the remaining 16 samples were stained using an anti-albumin allophycocyanin (APC)- conjugated antibody (188835) or corresponding isotype (20102) for 30 min at 4°C. Stained samples were acquired using a BD FACS Canto and analyzed in FlowJo v10.6.1 (BD Biosciences).

Conjugation of pan HLA-I antibody to Protein A Sepharose beads – 2.5 mg HLA-I antibody (w6/32) was added to 1 ml packed nProtein A Sepharose® 4 Fast Flow beads (GE Healhcare) at a concentration of 500 ug/ml in PBS with 1% Bovine serum albumin (BSA; Sigma) and incubated in suspension for 1 h rolling at RT. W6/32-bound beads were washed 3 times with 5 bead volumes PBS, 2 times with 5 bead volumes 0.2 M sodium borate buffer pH 9.0 and subsequently incubated for 30 min rolling at RT in 0.2 M sodium borate buffer pH 9.0, 16 mM dimethyl pimelimidate dihydrochloride (Sigma). Beads were washed once with 5 bead volumes 0.2 M ethanolamine pH 8.0 and incubated in 5 bead volumes 0.2 M ethanolamine pH 8.0 for 1 h rolling at RT. Beads were washed 2 times in 5 bead volumes 0.1 M glycine pH 2.5, 3 times in 5 bead volumes PBS, 1% BSA and stored at 4°C as 50% suspension in PBS, 0.1% sodium azide, 1% gelatin from cold water fish skin (Sigma).

Purification of HLA-I complexes – HLA-I peptidomes were obtained from 50-1800x10^6 patient- derived hepatocytes depending on availability. Cell pellets were thawed on wet ice in presence of 50 mM Tris pH 8.0, 150 mM NaCl, 5 mM EDTA, 1% gelatin from cold water fish skin, 1% BSA, 1 mM PMSF (Sigma), 1 tablet/5 ml cOmplete™ Mini EDTA-free

Protease Inhibitor Cocktail tablets (Roche), 1 tablet/10 ml PhosSTOP™ tablets (Roche) in ddH_2O (buffer A). Cells were lysed in a concentration of up to 200x10^6/ml for 1 h on wet ice in buffer A supplemented with 0.5% zwittergent 3-12 detergent (N-Dodecyl-N,N-dimethyl-3-ammonio-1-propanesulfonate; Sigma) (buffer B). Lysates were cleared by centrifugation at 4°C for 10 min at 17,000 g after which pellets were used for DNA isolation (see below). Supernatants were incubated with unconjugated nProtein A Sepharose® 4 Fast Flow beads in suspension for 1 h rolling at 4°C. Cleared lysates were subsequently incubated with w6/32 anti-HLA-I antibody covalently cross-linked to nProtein A Sepharose® 4 Fast Flow beads rolling for 1 h at 4°C in suspension after which HLA-I-bound beads were pelleted by centrifugation at 18 g for 30 sec at 4°C. HLA-I-bound beads were washed 3 times in 5 bead volumes of buffer B, 2 times in 5 bead volumes 20 mM Tris-Cl pH 8.0, 120 mM NaCl, once in 5 bead volumes 20 mM Tris-Cl pH 8.0, 1 M NaCl, 2 times in 5 bead volumes 20 mM Tris-Cl pH 8.0, 120 mM NaCl, once in 5 bead volumes PBS, 20 mM Tris-Cl pH 8.0 and once in PBS prior to peptide elution.

Immunoprecipitation efficiency by western blot – Denatured cleared lysate of 2.5x10^6 hepatocytes (in buffer B) was loaded on a 12% SDS Page gel and transferred to a PVDF membrane (Millipore). Membranes were incubated with an anti-HLA-I antibody (EMR8-5) and a polyclonal goat anti-mouse antibody. HLA-I was visualized using the Odyssey Image system and quantified using Image Studio Lite v5.2 (LI-COR).

Sample preparation and LC-MS/MS data acquisition – HLA peptides were eluted from the beads with 500 µl 0.15 % TFA at room temperature. This procedure was repeated four times to ensure complete elution. Eluted HLA peptides were lyophilized and stored at -20^0C. To separate HLA peptides from contaminating proteins the lyophilized peptides

were dissolved in 400 µl 0.1 % TFA and then filtered by centrifugation using a 10 kD MWCO column (Amicon, 42407). The filtered peptide fraction was desalted using an in-house fabricated 1 ml Sep-Pak column containing 10 mg C18 and 10 mg HLB resin. Peptides were eluted with 28 % acetonitrile / 0.1 % TFA and dried using vacuum centrifugation.

Nanoflow liquid chromatography tandem mass spectrometry (nanoLC-MS/MS) was performed on an EASY-nLC 1200 coupled to an Orbitrap Lumos Tribrid mass spectrometer (Thermo Fisher Scientific) operating in positive ion mode. Peptide mixtures were first trapped on a 2 cm x 100 µm Pepmap C18 column (Thermo Fisher Scientific, 164564) and then separated on an in-house packed 50 cm x 75 µm capillary column with 1.9 µm Reprosil-Pur C18 beads (Dr. Maisch) at a flow rate of 250 nL/min, using a linear gradient of 0–32 % acetonitrile (in 0.1% formic acid) during 120 min. MS spectra were acquired from 375 to 1,200 *m/z* in the Orbitrap at 120,000 resolution. Peptides were fragmented by HCD using a collision energy (CE) of 30 % and MS/MS spectra were subsequently recorded in the Orbitrap at 30,000 resolution.

For targeted proteomics, a parallel reaction monitoring (PRM) regime was used to select for a set of target peptides on an Orbitrap Eclipse Tribrid mass spectrometer (Thermo Fisher Scientific) operating in positive ion mode and running Tune version 3.3. The selected peptides were targets that had previously been detected in donor samples by conventional data-dependent acquisition (DDA) LC-MS/MS. Precursor peptides were selected in the quadrupole with an isolation width of 0.7 *m/z* and fragmented with HCD using 30 % CE. Fragmentation spectra were recorded in profile mode in the Orbitrap at

30,000 resolution. The maximum injection time was set to ‘dynamic’ with a minimum of 9 points measured across the chromatographic peak.

Bioinformatics analysis of DDA data– Mass spectrometry DDA data were analyzed with Peaks Studio v 10.5 (Bioinformatics Solutions Inc.) with the following parameters set: fragmentation mode: high energy CID (y and b ions); acquisition mode: DDA; MS Scan Mode and MS/MS Scan Mode: FT-ICR/Orbitrap; centroiding in MS/MS mode only; charge options 2-8. The PEAKS modules used were: 1) data refine, 2) de novo (parent mass error tolerance: 15 ppm; fragment mass error tolerance: 0.02 Da; enzyme: none); 3) Peaks (parent mass error tolerance: 15 ppm; fragment mass error tolerance: 0.02 Da; variable modifications: Oxidation (M) 15.99). The digest mode was set to ‘unspecific’ (no enzyme), while the error tolerances for parent mass and fragment masses were 10.0 ppm and 0.02 Da, respectively.

Gene ontology, KEGG pathway and STRING analysis – Gene name conversion, gene ontology (GO) and Kyoto Encyclopedia of Genes and Genomes (KEGG) analysis[1] was performed with the Homo sapiens background using DAVID Bioinformatics Resources v6.8[2] available at https://david.ncifcrf.gov/. For the purpose of GO and KEGG analysis, protein IDs were converted to gene names, which was successful in 96.5% of cases. Source proteins that were not recognised by the conversion tool were excluded from analysis. KEGG categorization was made according to the KEGG Pathway Database[3] available at https://www.genome.jp/kegg/pathway.html. A p-value < 0.05 after Bonferroni correction was considered significant. Visualization was performed using the R packages ‘GOplot1.0.2’[4] and ‘ggpubr0.4.0’ for GO and KEGG analysis, respectively. Analysis and visualization of protein-protein interactions was performed using the STRINGv11.0

webtool[5] available at https://string-db.org/. A minimal confidence level of 0.7 was used to identify protein-protein interactions.

Bioinformatics analysis of PRM data – All PRM MS data files were analyzed using the Skyline software suite. Chromatograms visualized by Skyline were manually reviewed, categorized and peak cutoffs assigned by Skyline were adjusted based on visual inspection. Five different categories of target peptides were defined based on the elution profiles: 0) No match; 1) Possibly related; 2) Unlikely match; 3) Possible match; 4) Good match; 5) Very Good match. Chromatograms marked as 'No match' showed no co-eluting fragment ion signals that could reasonably be considered to be the target of interest. Chromatograms in the 'Possibly related' category present co-eluting fragments, albeit with deviating relative intensity patterns from the library fragmentation spectrum. Chromatograms marked as 'Unlikely match' show a reduced number (but still at least two) of co-eluting fragments corresponding to the library spectrum. Chromatograms marked with 'Possible match' present >2 co-eluting fragment ions, albeit with slightly deviating intensity ratios compared to the library spectrum. Chromatograms marked as 'Good match' or 'Very Good match' present co-eluting fragment ion peaks corresponding to those in the library spectrum with (near to) complete sets of fragments with correct relative intensities. In contrast to those marked as 'Very Good match', chromatograms marked as 'Good match' have one or more minor issues that may seed some doubt as to their actual presence (e.g. fragment signals that did not properly overlap possibly as a result of low signals). Reports including fragment-level peak areas were exported from Skyline and processed together with the curation spreadsheet in R (version 4.0.0). Included fragment ion peak areas were summed per target in each separate LC-MS run. A final table

including the target sequence, summed peak area and the assigned category as described above was exported and included as Supplementary Information.

Immunohistochemistry – To determine the expression of surface and core HBV antigens in tumor (HCC) and non-tumorous tissue by automated IHC using the Ventana Benchmark ULTRA (Ventana Medical Systems Inc.), sequential 4 µm thick (FFPE) sections were stained for HBsAg and HBcAg using the Ultraview universal DAB detection Kit (Ventana). In brief, following deparaffinization and heat-induced antigen retrieval with CC1 (Ventana) for 64 min, the tissue samples were incubated with either anti-HBsAg or anti-HBcAg (table S2) for 32 min at 36˚C. Incubation was followed by hematoxylin II counter stain for 4 min and a blue coloring reagent for 4 min according to the manufactures instructions (Ventana).

Immunogenicity – Heparinized blood from hepatocyte donors was collected on the day of surgery. Buffy coats of healthy donors that previously cleared HBV infection and were seropositive for both anti-HBsAg and anti-HBcAg were obtained from Sanquin, the Netherlands. Peripheral blood mononuclear cells (PBMC) were obtained by Ficoll Paque Plus (GE Healthcare) density gradient centrifugation and cryopreserved in liquid nitrogen until use. Thawed PBMC were cultured and stimulated at 37°C in a humidified atmosphere supplemented with 5% CO_2. PBMC were stimulated at $1x10^6$/ml with a pool containing a maximum of 6 peptides of interest (Peptide2.0; >90% pure) at a concentration of 10 µg/ml/peptide in IMDM (Lonza) supplemented with 2% normal human AB serum (Sanquin), 1% ultraglutamin (Lonza), 1% penicillin/streptomycin (Gibco) and 50 IU/ml hIL-2 (Miltenyi). After 14 days, 200.000 cultured PBMCs were re-stimulated in duplicate for 48 h with each peptide of interest separately or corresponding controls. IFNγ

production was assessed by subjecting supernatants of re-stimulation cultures to the ELISA Max Standard Set Human IFNγ (BioLegend) according to the manufacturer's instructions. Absorbance was read at 450 nm using an Infinite 200 PRO microplate reader (Tecan). Peptide conditions with a mean OD value of at least the mean plus 3 times the standard deviation of the corresponding negative control were quantified. Conditions that did not meet this criterion were included as 0 pg/ml IFNγ produced. Quantification was performed by subtracting the average OD value of the negative control (measured in duplicate) from the average OD value of the peptide condition (measured in duplicate). Peptides against which IFNγ production was detected in at least one donor tested were classified as epitopes.

T cell fitness – For the CD3/CD28 activation assay, PBMC of 2 patients and 4 resolvers were thawed and resuspended in RPMI (Gibco) supplemented with 10% normal human AB serum (Sanquin), 2 mmol/L L-glutamine (Lonza) and 1% penicillin/streptomycin (Gibco). 100.000 PBMC were seeded in each well of a 96-well round-bottom culture plate and stimulated with 25ng/ml of anti-CD3 (OKT3) and 1µg/ml of anti-CD28 (CD28.2) in triplicate. PBMC were harvested after 24 h and 72 h of stimulation and subjected to antibody staining for CD3 (CD3-FITC, clone UCHT1), CD4 (CD4-PerCP-eFluor 710, clone RPA-T4), CD8 (CD8-SB645, clone OKT8), CD45 (CD45-APC, clone HI30), CD69 (CD69-PE, clone TP1.55.3), CD25 (CD25-PECY7, clone BC96), CD137 (CD137-BV421, clone 4B4-1), HLA-DR (HLA-DR-BV605, clone L243), Ki67 (Ki-67-APC, clone 20Raj1). T-cell proliferation and upregulation of activation markers was determined based on Ki67-expression and CD69, CD25, CD137 and HLA-DR expression on a FACSymphony A3 4-Laser Cell Analyzer (BD Biosciences) and analyzed using FlowJo software v10.6.1 (BD

Biosciences). Viability of cells was assessed using Fixable Viability Dye eFluor™ 506 (Thermo Fisher Scientific). Fixation and permeabilization were performed using the Foxp3/ Transcription Factor Staining Buffer Set (Thermo Fisher Scientific) according to manufacturer's instructions. In addition, appropriate isotype matched control antibodies were used (i.e. IgG2b-PE clone 27-35; IgG1-PECY7 clone MOPC-21; IgG1-BV421 clone X40; IgG2a-BV605 clone MOPC-173).

HLA typing – DNA isolation was performed on nuclear pellets obtained during HLA-I purification (hepatocyte donors) or on PBMC (HBV resolvers) using the QIAamp DNA Mini kit (Qiagen) according to manufacturer's instructions. HLA typing of hepatocyte donors was performed by the Institute for immunology and Infectious Diseases (Murdoch, Australia) using Illumina NGS. HLA-typing of HBV resolver blood donors was performed by the in-house department of Internal Medicine (Rotterdam, the Netherlands) using the Illumina GSA beadchip GSA MD v2.

SUPPLEMENTARY FIGURES

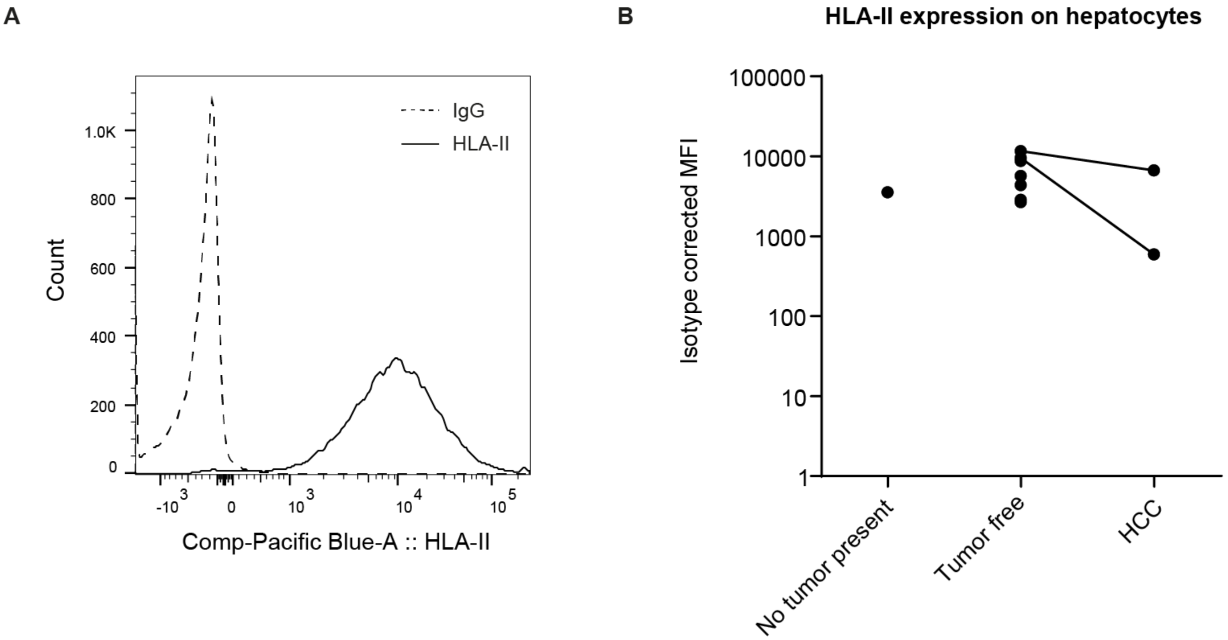

Fig. S1. HLA-II expression on primary hepatocytes. (A) Flow cytometric staining of HLA-II on albumin positive hepatocytes in a representative histogram and (B) across tissue types.

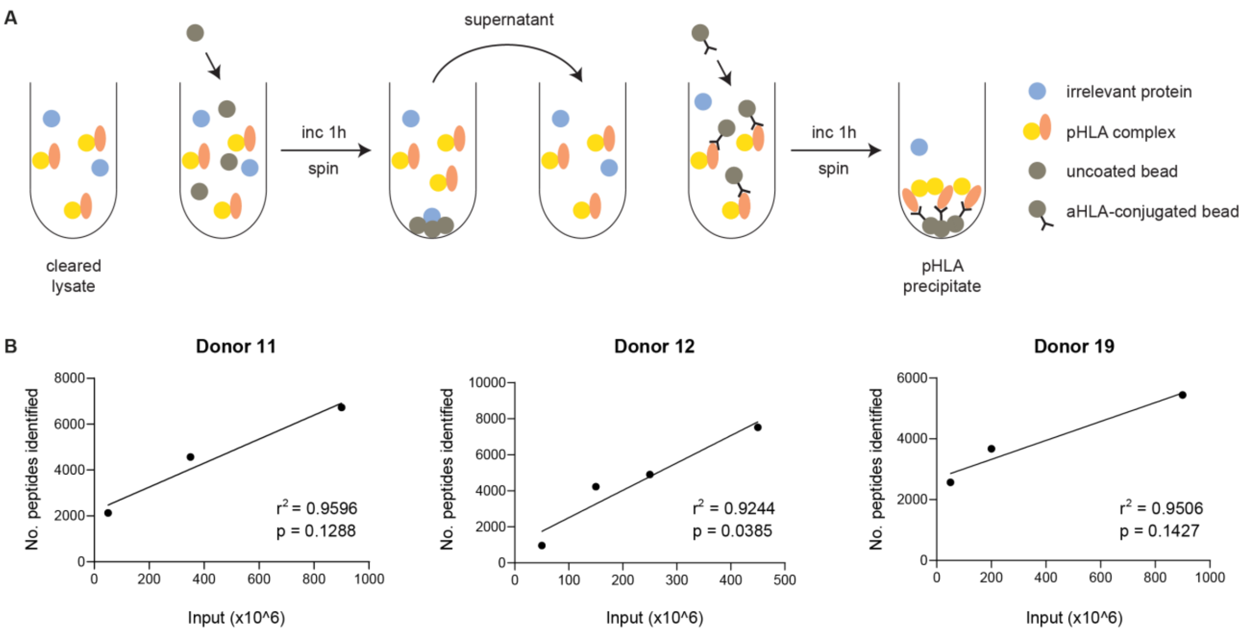

Fig. S2. HLA-I immunoprecipitation. (A) The procedure by which HLA-I was precipitated. Cleared cell lysates are incubated with uncoated sepharose beads to reduce background and subsequently incubated with sepharose beads crosslinked to pan HLA-I (w6/32) antibody. (B) Relation between absolute number of peptides identified and cellular input within tumor free tissues of 3 independent donors as analyzed by simple linear regression.

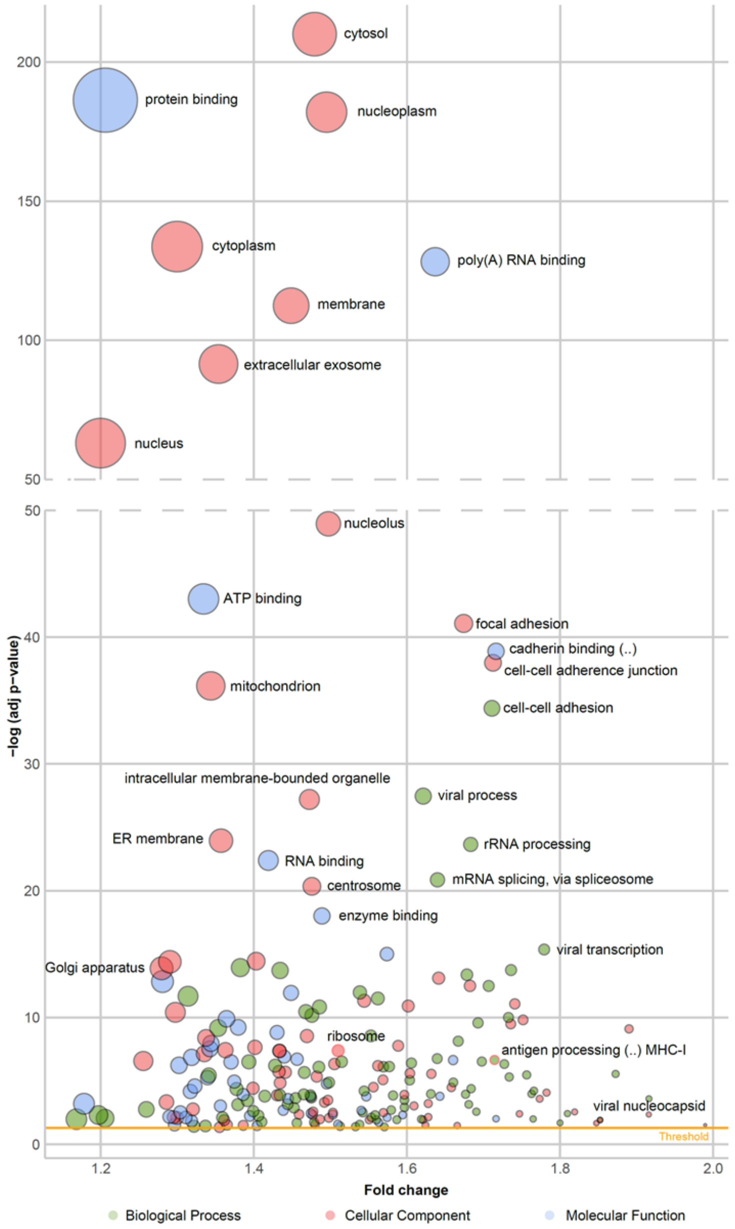

Fig. S3. HLA-I peptide localization and function. Significantly enriched GO terms in all identified source proteins relative to the human genome. Balloon color represents GO term classification as indicated. Size of the balloon represents the number of proteins annotated within the respective GO-term. Balloons are positioned according to the –log of the Bonferroni corrected p-value (fisher exact test; y-axis) and the fold change relative to the human genome (x-axis). The threshold for significance is set at p=0.05 after Bonferroni correction (yellow line).

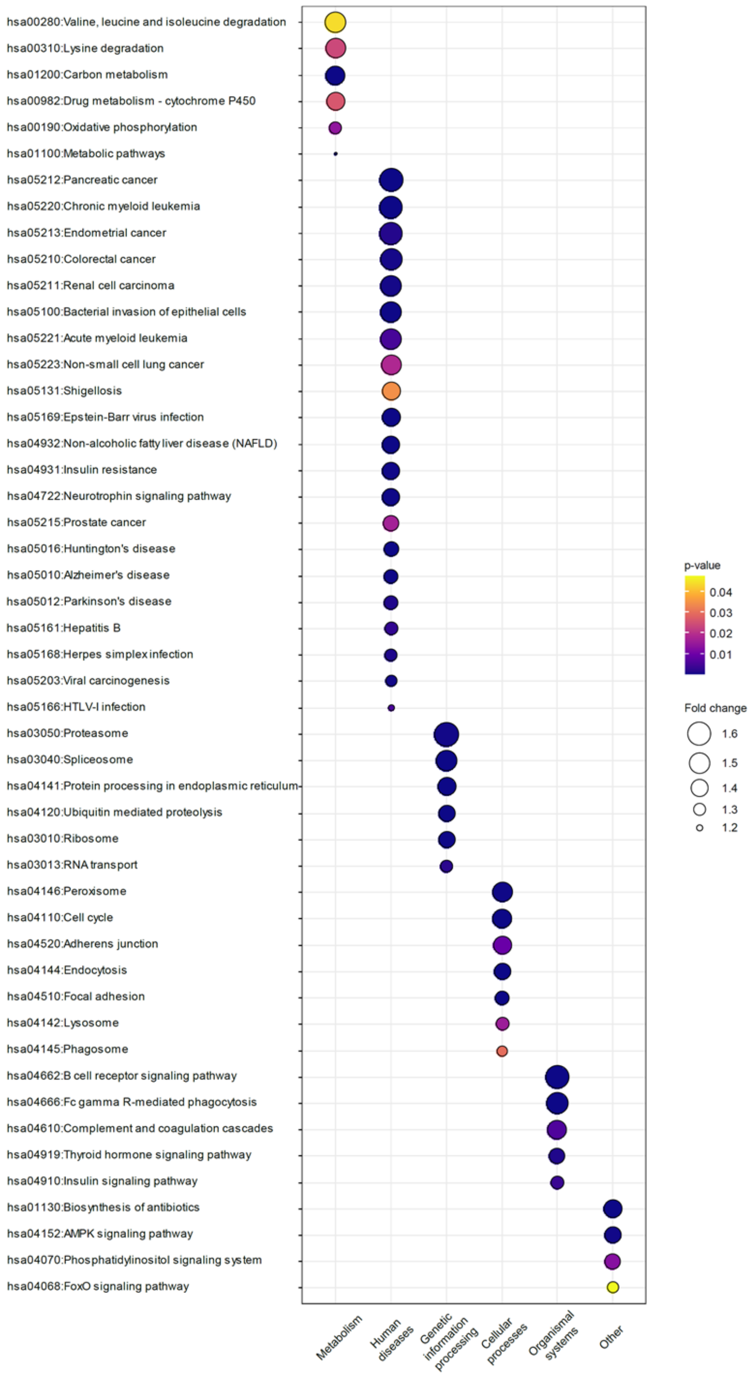

Fig. S4. HLA-I peptide functional annotation. KEGG pathways that are significantly enriched in identified source proteins relative to the human genome (rows). Pathways are categorized as indicated (columns). Within categories, pathways are sorted based on fold enrichment over the human genome (balloon size). Balloon color represents the Bonferroni corrected p-value of a fisher's exact test.

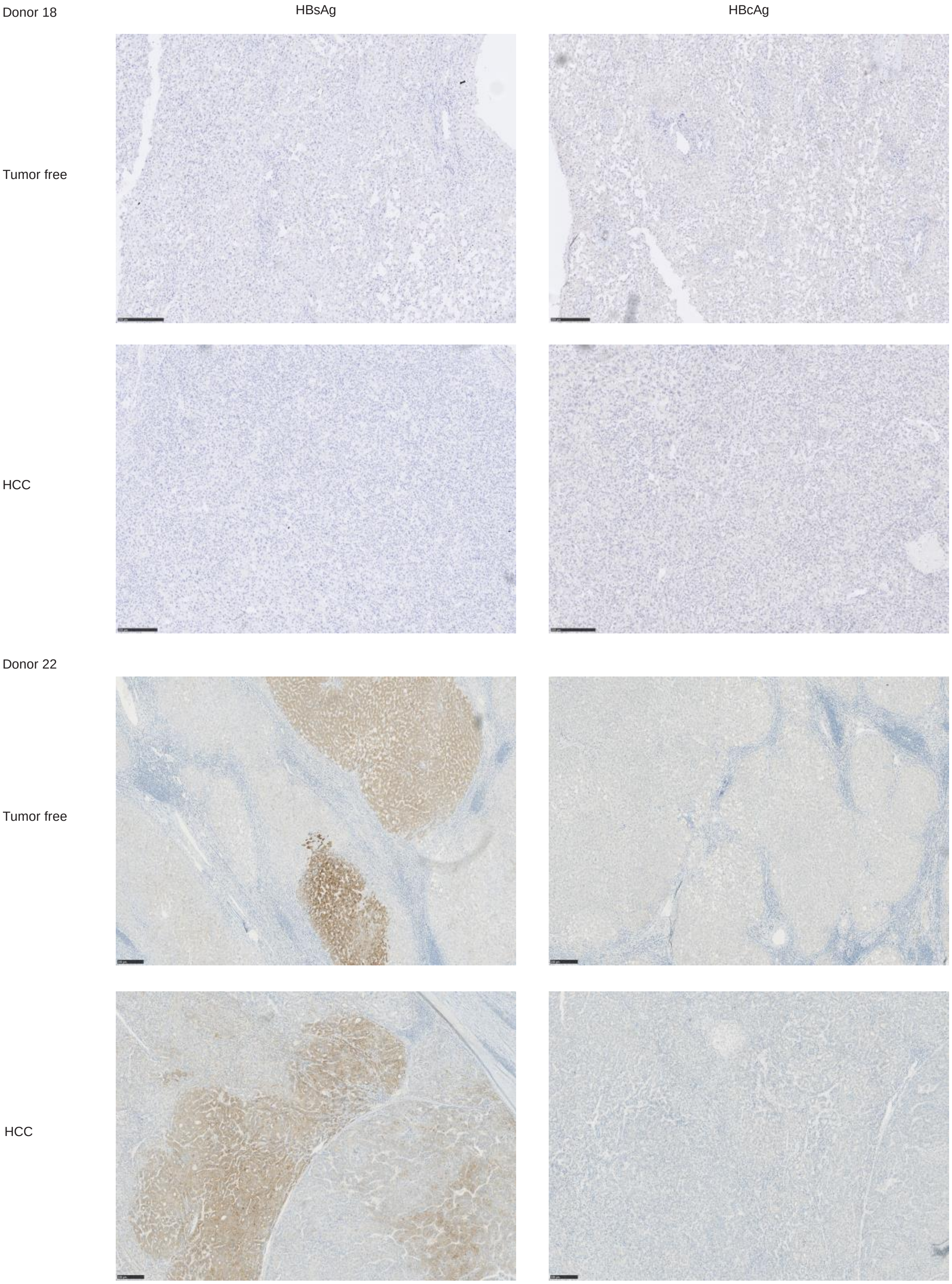

Fig. S5. Immunohistochemistry of liver tissues from donors with (a clinical history of) HBV infection. Representative stainings of HBsAg (left) and HBcAg (right) in donors 18 (above) and 22 (below). The scale bar indicates 250 µm.

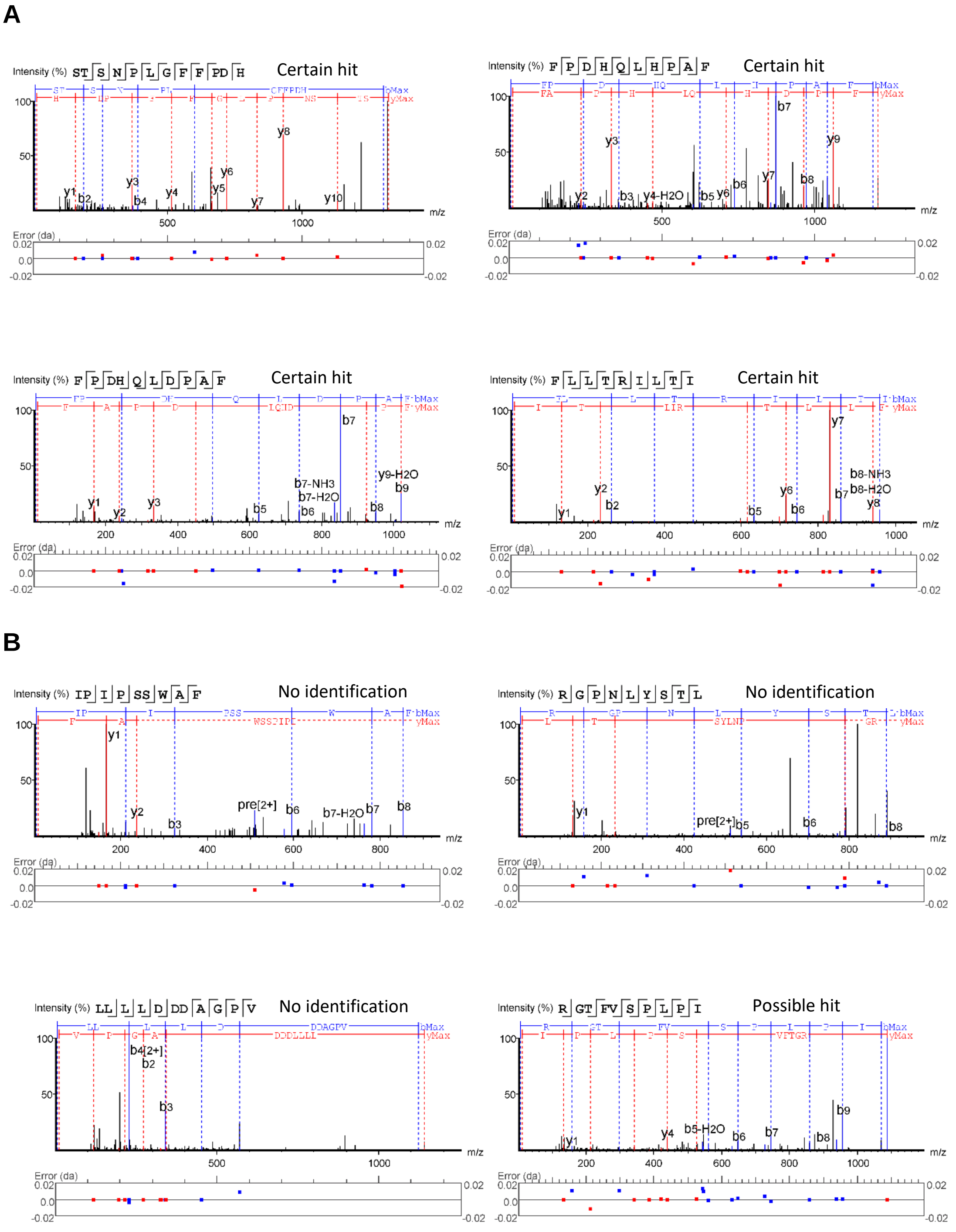

Fig. S6. MS/MS spectra of HBV-derived peptides. MS/MS spectra of all HBV-derived peptides that are identified by DDA as listed in table 2. Spectra were of (A) sufficient or (B) insufficient quality for downstream PRM analysis.

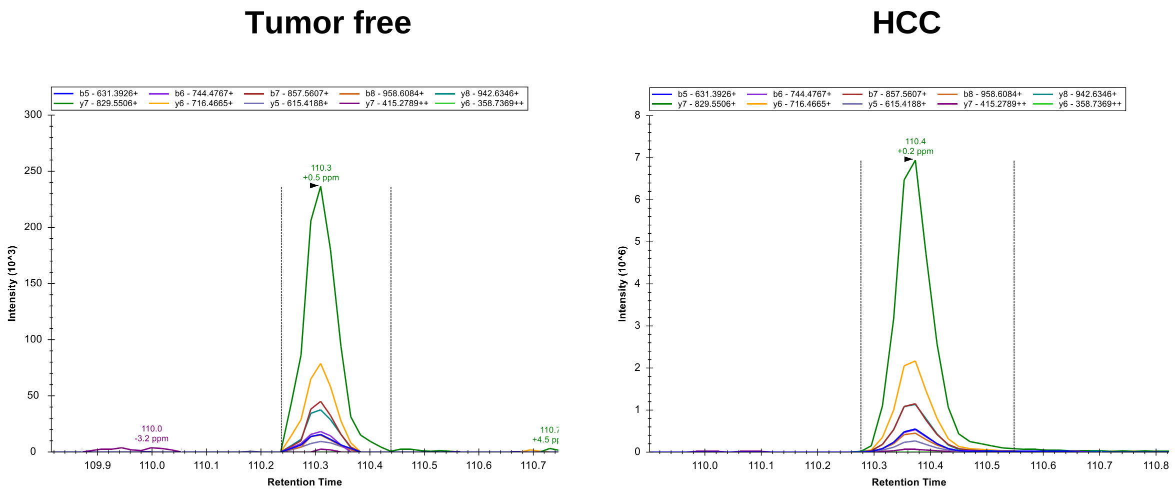

Fig. S7. Examples of category 5 'very good match' PRM chromatograms. Chromatograms of peptide FLLTRILTI ($HBsAg_{183-191}$) in tumor free (left) and HCC (right) samples from patient 22.

A

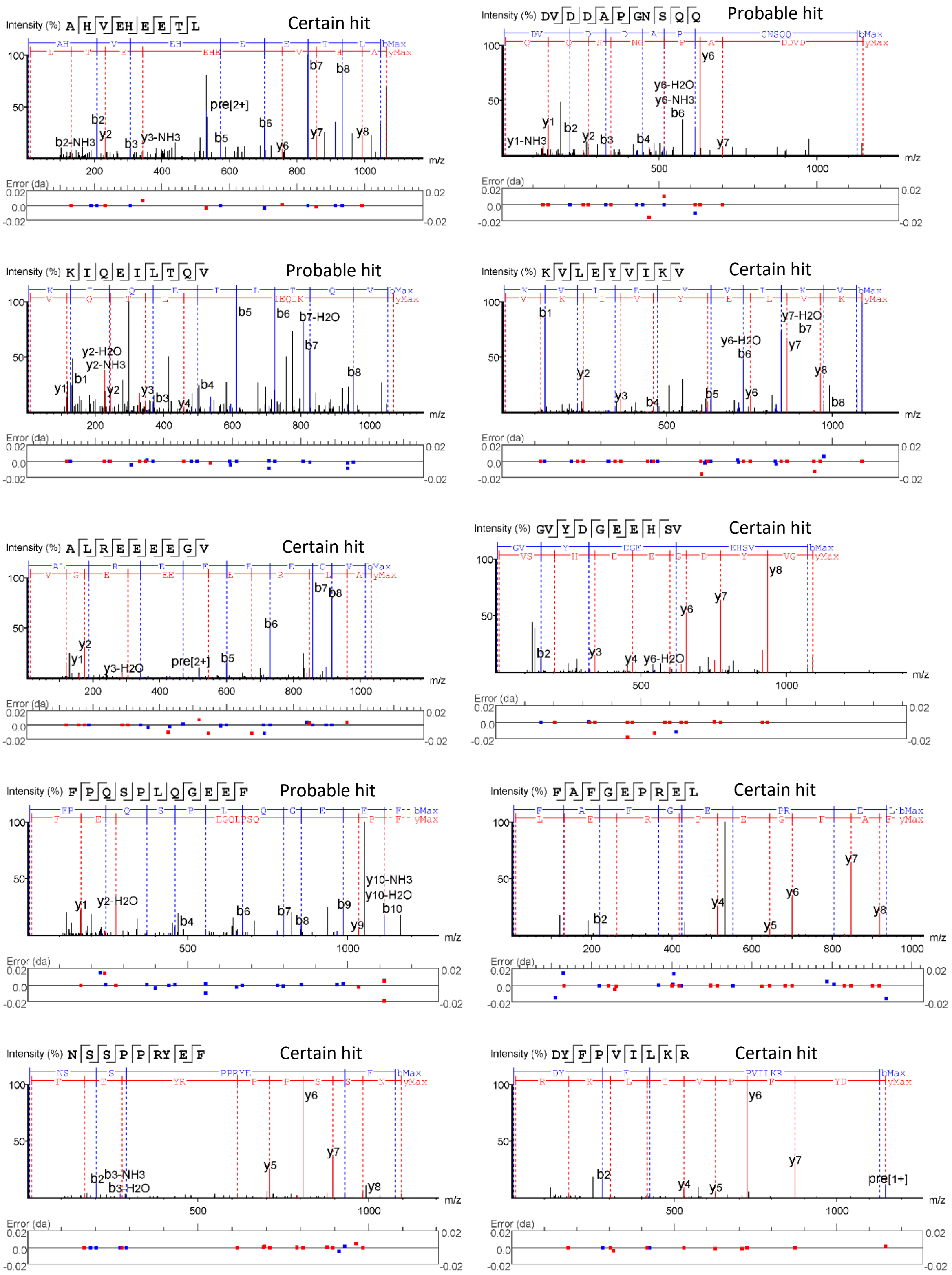
Intensity (%) A H V EH E E T L
Certain hit
Intensity (%) DV D D A P GN S Q Q
Probable hit
Intensity (%) K I Q E I L T Q V
Probable hit
Intensity (%) K V L E Y V I K V
Certain hit
Intensity (%) A L R E E E E G V
Certain hit
Intensity (%) GV Y D G E E H SV
Certain hit
Intensity (%) F P Q S P L Q G E E F
Probable hit
Intensity (%) F A F G E P R E L
Certain hit
Intensity (%) N S S P P RY E F
Certain hit
Intensity (%) DY F P V I L K R
Certain hit
m/z
Error (da)

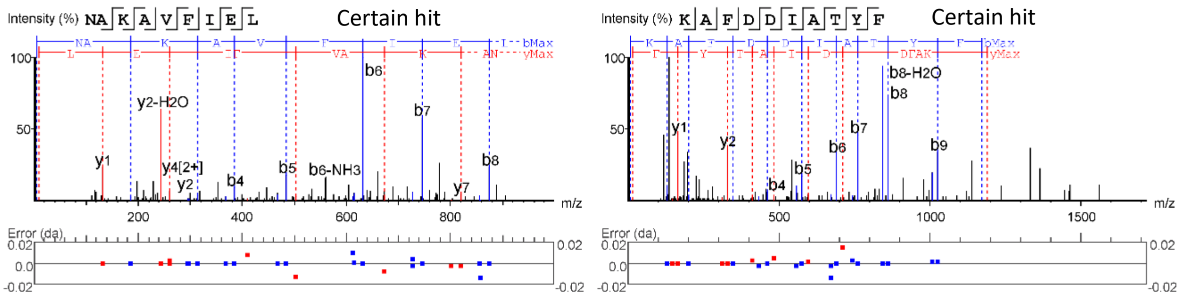

B

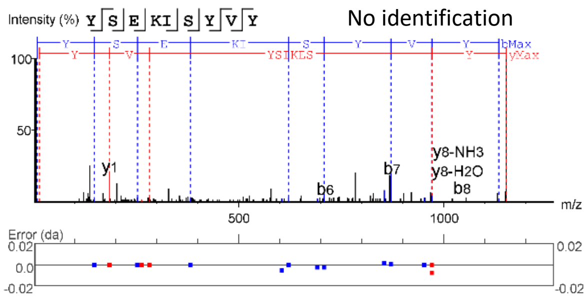

Fig. S8. MS/MS spectra of all 9-11-mer TAA-derived peptides. MS/MS spectra of all TAA-derived 9-11mers that are exclusively identified in HCC eluates by DDA as listed in table 3. Spectra were of (A) sufficient or (B) insufficient quality for downstream PRM analysis.

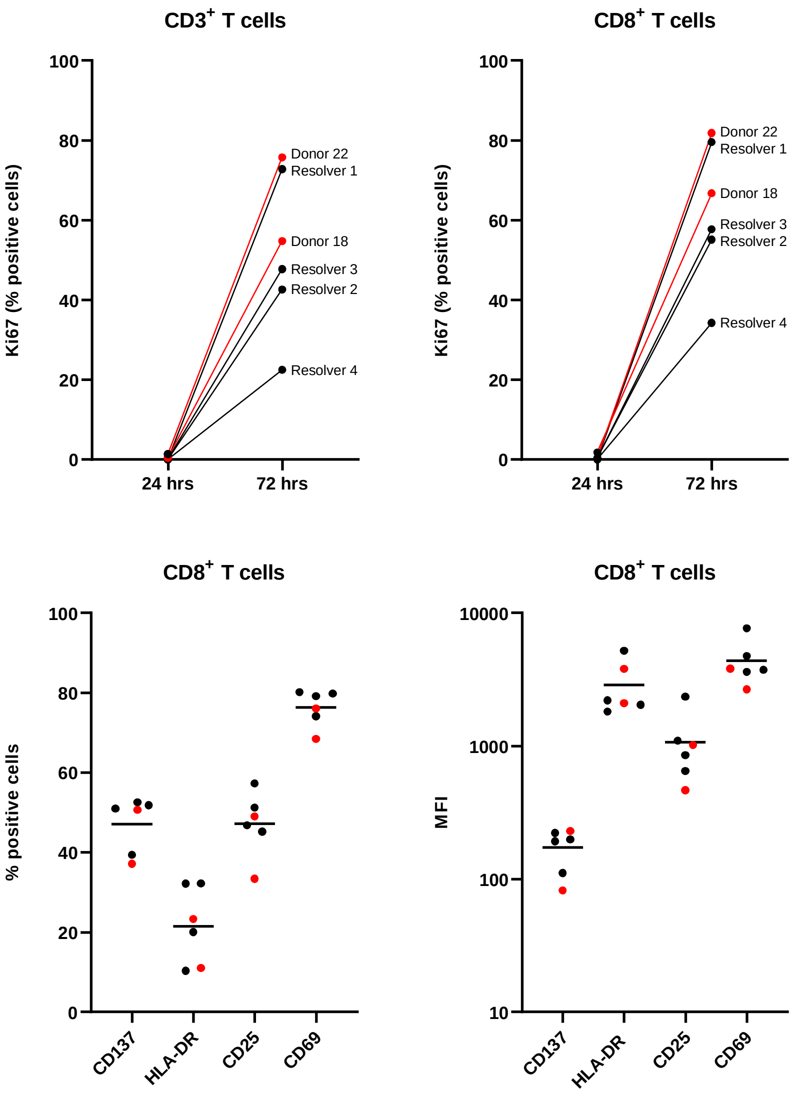

Fig. S9. Overall fitness of peripheral T cells from HBV resolvers and patients. PBMCs of patients (red) and resolvers (black) that were used for peptide immunogenicity testing (figure 4) were also exposed to CD3/CD28 stimulation for either 24 or 72 hours. Subsequently, cells were labeled for T cell identification and activation markers. The upper two plots depict the proliferation (% Ki67$^+$ cells) of total CD3$^+$ T cells (left) and CD3$^+$CD8$^+$ T cells (right) after 24 and 72 hours following CD3/CD28 stimulation. The lower two graphs depict expression of activation markers within CD3$^+$CD8$^+$ T cells in percentage of marker positive cells (left) and as MFI of indicated markers (right) after 24 hours of stimulation.

SUPPLEMENTARY TABLES

Supplementary Table 1. HLA-I types of hepatocyte donors							
Donor	HLA-A		HLA-B		HLA-C		Irrelevant HLA-A
1	01:01	03:01	07:02	15:01	04:01	07:02	02:01
2	02:02	33:01	14:02	53:01	04:01	08:02	01:01
3	02:01	03:01	07:02	44:02	07:02	07:04	24:02
4	02:01	30:01	35:01[a]	42:01[b]	16:01	17:01	24:02
5	01:01	24:02	13:02	27:05	01:02	06:02	03:01
6	24:02	26:01	15:01	38:01	03:03	12:03	02:01
7	24:02	68:01	07:05	39:01	07:02	15:05	02:01
8	02:01	11:01	15:01[c]	35:01[d]	03:04	04:01	24:02
9	02:01	03:01	15:01	37:01	03:04	06:02	24:02
10	23:01	26:01	08:01	15:03	02:10	03:04	02:01
11	24:02	30:01	13:02	27:07	06:02	15:02	03:01
12	02:01	33:03	15:01	44:03	01:02	07:01	24:02
13	29:01	31:01	35:02	39:01	04:01	12:03	02:01
14	02:01	24:02	51:01	55:01	01:02[e]	03:03[f]	03:01
15	23:01	30:01	35:01	53:01	04:01	04:01	02:01
16	24:02	24:02	18:01	38:02	07:01[g]	07:02[h]	02:01
17	01:01	02:01	08:01	14:01	07:01	08:02	03:01
18	02:01	02:01	44:02	57:01	04:01	07:04	03:01
19	33:01	68:02	52:01	78:01	16:01	16:01	24:02
20	02:01	32:01	37:01	44:02	05:01	06:02	24:02
21	02:01	11:01	14:01	51:01	08:02	15:02	24:02
22	01:01	01:01	08:01	35:02	04:01	07:01	24:02
[a]35:01 or 07:235							
[b]42:01 or 35:87							
[c]15:01 or 15:20							
[d]35:01 or 35:43							
[e]01:02 or 01:131							
[f]03:03 or 03:86							
[g]07:01 or 07:19							
[h]07:02 or 07:27							

Supplementary Table 2. Antibodies used for immunohistochemistry					
**Target**	**Type**	**Concentration**	**Company**	**Clone**	**Lot number**
HBsAg	mouse	1/50	Thermo Fisher Scientific	T9	31452004C
HBcAg	rabbit	1/75	Neomarkers	AB-1	1413A1510J

Supplementary Table 3. Immunohistochemistry of liver tissues from donors with (a clinical history of) HBV infection				
**Donor**	**Tumor free**		**HCC**	
	**HBsAg**	**HBcAg**	**HBsAg**	**HBcAg**
2	+	-	NA	NA
3	+	-	NA	NA
4	-	-	NA	NA
5	+	-	NA	NA
6	-	-	NA	NA
7	+	-	-	-
10	-	-	-	-
11	+	-	-	-
12	+	-	-	-
13	-	-	NA	NA
14	+ (focal)	-	NA	NA
15	+	-	NA	NA
16	+	-	-	-
17	-	-	-	-
18	-	-	-	-
19	+	-	-	-
20	+	+	+	-
21	-	-	-	-
22	+	-	+	-
NA: not applicable				

Supplementary Table 4. Detailed PRM results for HBV-derived 9-11mers to which PRM was applied										
Protein	Position	Sequence	Spectrum match manually confirmed?	Summed peak area	PRM category	Donor	Tissue	HBV status	Relation to DDA result	Mapping to HLA in donor
HBsAg	183-191	FLLTRILTI	Certain	118996	(5) Very Good Match	14	TF	C	Other donor	A*02:01; B*51:01; C*01:02; C*03:03
				183028	(5) Very Good Match	11	TF	C	Other donor	B*13:02; C*06:02; C*15:02
				1845450	(5) Very Good Match	22	TF	AC	Same donor, other sample	B*08:01; C*04:01; C*07:01
				60018956	(5) Very Good Match	22	HCC	AC	Same donor, same sample	B*08:01; C*04:01; C*07:01
				241228	(4) Good Match	12	TF	C	Other donor	A*02:01; C*01:02; C*07:01
HBsAg	14-23D	FPDHQLDPAF	Certain	95889357	(5) Very Good Match	22	HCC	AC	Same donor, same sample	B*35:02; C*04:01
				64135370	(5) Very Good Match	5	NTP	C	Other donor	No
				59403779	(5) Very Good Match	22	TF	AC	Same donor, other sample	B*35:02; C*04:01
HBsAg	14-23H	FPDHQLHPAF	Certain	29890007	(5) Very Good Match	22	HCC	AC	Same donor, same sample	B*08:01; B*35:02; C*04:01
Tissue: HCC = Tumor; TF = Tumor Free; NTP = No Tumor Present										

Supplementary Table 5. Peptides derived from known tumor-associated antigens that are identified in HLA-I eluates from benign tissue types						
			Donor samples in which peptides are identified			
Antigen	Position	Sequence	Non-diseased	No tumor present	Tumor free[a]	HCC
BRDT	203-211	TAAQVTKGV				19
BRDT	259-267	QQYNVVKTV				19
BRDT	532-540	RVVHIIQSR			13	
BRDT	608-616	DVNNQLNSR				19
CCDC110	270-281	QTDPDVHRNGKY		5	22	22
CEP55	197-205	GLLAKIFEL			17	
CEP55	275-284	EVHNLNQLLY			10	
DCAF1	42-49	IEKETEEY			16	16
DCAF1	137-146	READQPLRTY		3	12, 12, 12, 18, 20	18
DCAF1	291-299	DPDRMFVEL			13, 22	22
DCAF1	325-333	IEQRLILQY			16	16
DCAF1	326-333	EQRLILQY	1		8, 9, 12, 12	
DCAF1	438-486	DRYDGLRRL		2	17, 21	17, 21
DCAF1	531-539	HLAIKLEQV		3	9, 14, 17, 18, 20	17, 18, 20
DCAF1	628-636	AILTVVPKI			9, 14, 18	17, 18
DCAF1	677-684	IQKSALQI				19
DCAF1	808-816	YAAELIERV			19	19
DCAF1	816-823	VSGKPLLI			21	
DCAF1	837-845	VVAQSRISF	1	6	8, 9, 12, 12, 12	
DCAF1	951-959	SPLIGRISF			7	
DCAF1	987-994	SPAIKKQL			10, 22	22
DCAF1	1046-1054	APINFTSRL		3		
DCAF1	1115-1122	GQLKLYNV			11	
DCAF1	1115-1123	GQLKLYNVF	1		12	
IL13RA1	268-276	QTETHNVFY			17	17
KNL1	313-321	HTANIQTLI				19
KNL1	692-700	IATSHNIVY			19	19
KNL1	792-800	KSHTVVIGF				18
KNL1	837-845	SPIEKSGVL	1	3		
KNL1	1463-1471	IYVIPQPHF			10	
KNL1	2308-2316	DIATILSKV			19, 19	19
LDHC	3-11	TVKEQLIEK		4		
NUF2	10-18	NVAEIVIHI			19, 19, 19	19

NUF2	56-64	VYGIRLEHF				16
NUF2	119-127	RFLSGIINF			10	
NUF2	172-180	VPVEEQEEF		4		
NUF2	271-279	EVVEKYEIY			10	
NUF2	299-309	KIQDLSDNREK				21
NUF2	353-362	ATAQFKINKK			8	
SPA17	66-74	DRFYNNHAF			17	
SPA17	115-123	EVAAVKIQA				19
SPAG9	87-95	TQYEREKAL				21
SPAG9	137-145	NYADQISRL			14	
SPAG9	766-773	SATKVLII			14	
SPAG9	917-925	HVFTDPLGV			11	
SPAG9	942-950	DAYKDQISV			14	19
SPAG9	1019-1027	DGTLAIFHR			19, 19	19
SPAG9	1083-1093	DAHPRKESQVR				19
SPAG9	1115-1023	YHAHTYQHL		6		
SPAG9	1143-1151	FSFVRITAL		6	8, 19	19
SPAG9	1224-1231	DAVKFFVA			19	
SPAG9	1224-1232	DAVKFFVAV			14, 19, 19, 19, 21	19, 21
TTK	821-829	SPNSILKAA			14	

[a]Donor numbers of tumor free tissues used for titration experiments are listed several times when a peptide is identified repeatedly in independent sampling of the same tissue

Supplementary Table 6. Detailed PRM results for TAA-derived 9-11mers to which PRM was applied									
**Protein**	**Position**	**Sequence**	**Spectrum match manually confirmed?**	**Summed peak area**	**PRM category**	**Donor**	**Tissue**	**Relation to DDA result**	**Mapping to HLA in donor**
GPC3	376-384	AHVEHEETL	Certain	11416128	(5) Very Good Match	16	HCC	Same donor, same sample	B*38:02; C*07:01; C*07:02
GPC3	531-541	DVDDAPGNSQQ	Possible	3545702	(5) Very Good Match	21	HCC	Same donor, same sample	No
IGF2BP3	552-560	KIQEILTQV	Possible	13258530	(5) Very Good Match	17	HCC	Same donor, same sample	A*02:01
				3873224	(5) Very Good Match	20	HCC	Other donor	A*02:01; A*32:01; C*05:01 C*06:02
				2480442	(4) Good Match	4	NTP	Other donor	A*02:01; A*30:01; C*17:01
MAGEA1	278-286	KVLEYVIKV	Certain	12489365	(5) Very Good Match	17	HCC	Same donor, same sample	A*02:01; C*07:01
				992245	(5) Very Good Match	18	HCC	Other donor	A*02:01; C*04:01; C*07:04
				1452185	(5) Very Good Match	6	NTP	Other donor	C*12:03
MAGEA1	301-309	ALREEEEGV	Certain	17125355	(5) Very Good Match	17	HCC	Same donor, same sample	A*02:01
MAGEB2	231-240	GVYDGEEHSV	Certain	20233257	(5) Very Good Match	18	HCC	Same donor, same sample	A*02:01; C*04:01
MAGEC1	611-621	FPQSPLQGEEF	Possible	14079269	(5) Very Good Match	22	HCC	Same donor, same sample	B*35:02; C*04:01
MAGEC1	1035-1043	FAFGEPREL	Certain	524830012	(5) Very Good Match	19	HCC	Same donor, same sample	A*68:02; B*52:01; B*78:01; C*16:01
MAGEC1	1061-1069	NSSPPRYEF	Certain	53280673	(5) Very Good Match	19	HCC	Same donor, same sample	A*68:02; B*52:01; B*78:01; C*16:01
MAGEC2	176-184	DYFPVILKR$^{(++/+++)}$	Certain	72097015(++) 48265970(+++)	(5) Very Good Match	19	HCC	Same donor, same sample	A*33:01
PNMA5	61-69	NAKAVFIEL	Certain	182776750	(4) Good Match	19	HCC	Same donor, same sample	A*68:02; B*52:01; B*78:01; C*16:01
SSX1	23-32	KAFDDIATYF	Certain	18694155	(5) Very Good Match	18	HCC	Same donor, same sample	B*57:01; C*04:01; C*07:04

Supplementary Table 7. HLA-I types of HBV resolved blood donors						
Resolver	HLA-A		HLA-B		HLA-C	
1	01:01	02:01	08:01	40:01	07:01	03:04
2	01:01	24:02	08:01	07:02	07:01	07:02
3	01:01	03:01	08:01	13:02	07:01	06:02
4	01:01	02:01	08:01	38:01	07:01	12:03

Supplementary refereces

1 Kanehisa M, Goto S, Sato Y, Furumichi M, Tanabe M. KEGG for integration and interpretation of large-scale molecular data sets. *Nucleic Acids Res* 2012;**40**:109–14. doi:10.1093/nar/gkr988

2 Huang DW, Sherman BT, Lempicki RA. Systematic and integrative analysis of large gene lists using DAVID bioinformatics resources. *Nat Protoc* 2009;**4**:44–57. doi:10.1038/nprot.2008.211

3 Kanehisa Laboratories. KEGG Pathway Database. Last Updat. March 10, 2020. https://www.genome.jp/kegg/pathway.html

4 Walter W, Sánchez-Cabo F, Ricote M. GOplot: An R package for visually combining expression data with functional analysis. *Bioinformatics* 2015;**31**:2912–4. doi:10.1093/bioinformatics/btv300

5 Szklarczyk D, Gable AL, Lyon D, Junge A, Wyder S, Huerta-Cepas J, *et al.* STRING v11: Protein-protein association networks with increased coverage, supporting functional discovery in genome-wide experimental datasets. *Nucleic Acids Res* 2019;**47**:D607–13. doi:10.1093/nar/gky1131
