# Supplementary material for: Immunopeptidome of hepatocytes isolated from patients with HBV infection and hepatocellular carcinoma
Source: JHEP Rep. 2022 Sep 5;4(11):100576. doi: 10.1016/j.jhepr.2022.100576 (PMC9523389; doi:10.1016/j.jhepr.2022.100576)
Supplement: Multimedia component 2 [file mmc2.docx]

**JHEP Reports**

**CTAT methods**

Tables for a “Complete, Transparent, Accurate and Timely account” (CTAT) are now mandatory for all revised submissions. The aim is to enhance the reproducibility of methods.

- Only include the parts relevant to your study
- Refer to the CTAT in the main text as ‘Supplementary CTAT Table’
- Do not add subheadings
- Add as many rows as needed to include all information
- Only include one item per row

**If the CTAT form is not relevant to your study, please outline the reasons why:**

| NA |
| --- |

- 1. **Antibodies**

| **Name** | **Citation** | **Supplier** | **Cat no.** | **Clone no.** |
| --- | --- | --- | --- | --- |
| CD235a, Glycophorin A | Kondratov et al., J Extracell Vesicles. 2020 Mar 30;9(1):1743139 | Dako | R7078 | JC159 |
| Pacific Blue™ anti-human HLA-A,B,C Antibody | Lo et al., Cell Stem Cell. 2021 Jun 3;28(6):1023-1039.e13 | BioLegend | 311418 | w6/32 |
| Pacific Blue™ Mouse IgG2a, κ Isotype Ctrl Antibody | Kalinski et al., Elife. 2020 Dec 2;9:e60223 | BioLegend | 400235 | MOPC-173 |
| BV421 Mouse Anti-Human HLA-DR, DP, DQ | Ziegler et al., Immunobiology. 1986; 171(1-2):77-92 | BD Biosciences | 564244 | Tu39 |
| BV421 Mouse IgG2a, k Isotype Control | NA | BD Biosciences | 562439 | G155-178 |
| CD45 Monoclonal Antibody (HI30), APC, eBioscience™ | Hajizadeh et al., Iran Endod J. 2018 Winter;13(1):94-101 | eBioscience | 17-0459-42 | HI30 |
| CD45 Monoclonal Antibody (HI30), APC-eFluor™ 780, eBioscience™ | Combes et al., Nature. 2021 Mar;591(7848):124-130 | eBioscience | 47-0459-42 | HI30 |
| CD45-PE, J33 | NA | Beckman Coulter | IM2078U | J33 |
| ALB Antibody (N-18) | Suzuki et al., Int J Mol Sci. 2020 Dec 7;21(23):9319 | Santa Cruz Biotech | Sc-46291 | Polyclonal |
| Normal Goat IgG Biotinylated Control | Martinez-Gallo et al., J Allergy Clin Immunol. 2013 Feb;131(2):468-76 | R&D systems | BAF108 | Polyclonal |
| Donkey anti-Goat IgG (H+L) Cross-Adsorbed Secondary Antibody, Alexa Fluor 647 | Ryosaka et al., STAR Protoc. 2022 Jun 17;3(3):101484 | Thermo Fisher Scientific | A-21447 | Polyclonal |
| Human Serum Albumin APC-conjugated Antibody | Pettinato et al., Sci Adv. 2021 Aug 18;7(34):eabj2800 | R&D systems | IC1455A | 188835 |
| Mouse IgG2A APC-conjugated Antibody | Zhu et al., Oncol Lett. 2021 Jun;21(6):481. | R&D systems | IC003A | 20102 |
| Ultra-LEAF™ Purified anti-human HLA-A,B,C Antibody | Dangaj et al., Cancer Cell. 2019 Jun 10;35(6):885-900.e10. | BioLegend | 311442 | W6/32 |
| Anti-HLA Class 1 ABC antibody [EMR8-5] | Mints et al., Sci Rep. 2021 Jan 19;11(1):1782 | Abcam | Ab70328 | EMR8-5 |
| IRDye® 800CW Goat anti-Mouse IgG Secondary Antibody | NA | LI-COR | 926-32210 | Polyclonal |
| Hepatitis B Virus Surface Mouse anti-Virus, Clone: T9, Invitrogen™ | NA | Thermo Fisher Scientific | MA5-13059 | T9 |
| Hepatitis B Virus Core Antigen (HBVcAg) Ab-1 | Stahl et al., Proc Natl Acad Sci USA, 1982 79:1606. | Neomarkers | RB-1413 | AB-1 |
| Ultra-LEAF™ Purified anti-human CD3 Antibody | Campos Carrascosa et al., J Immunother Cancer. 2020 Sep;8(2):e000816 | BioLegend | 317326 | OKT3 |
| Ultra-LEAF™ Purified anti-human CD28 Antibody | Wang et al., Cell Stem Cell. 2021 Sep 2;28(9):1597-1613.e7 | BioLegend | 302934 | CD28.2 |
| CD3-FITC, UCHT1, 100 Tests, CE | NA | Beckman Coulter | A07746 | UCHT1 |
| CD4 Monoclonal Antibody (RPA-T4), PerCP-eFluor™ 710, eBioscience™ | Prat et al., Cancers (Basel). 2021 Apr 13;13(8):1845. | Thermo Fisher Scientific | 46-0049-42 | RPA-T4 |
| CD8a Monoclonal Antibody (OKT8 (OKT-8)), Super Bright™ 645, eBioscience™ | Clairfeuille et al., J Biol Chem. 2015 Jun 5;290(23):14504-17 | Thermo Fisher Scientific | 64-0086-42 | OKT8 |
| CD69-PE, TP1.55.3, 2 mL, ASR | NA | Beckman Coulter | IM1943U | TP1.55.3 |
| CD25 Monoclonal Antibody (BC96), PE-Cyanine7, eBioscience™ | Catakovic et al., Oncoimmunology. 2017 Sep 21;7(1):e1371399 | Thermo Fisher Scientific | 25-0259-42 | BC96 |
| Brilliant Violet 421™ anti-human CD137 (4-1BB) Antibody | Wenthe et al., Cancer Immunol Immunother. 2021 Oct;70(10):2851-2865. | BioLegend | 309820 | 4B4-1 |
| Brilliant Violet 605™ anti-human HLA-DR Antibody | Hagen et al., Cell Rep. 2020 Dec 8;33(10):108485 | BioLegend | 307640 | L243 |
| Ki-67 Monoclonal Antibody (20Raj1), APC, eBioscience™ | Gaudreau et al., J Thorac Oncol. 2021 Jan;16(1):127-139 | Thermo Fisher Scientific | 17-5699-42 | 20Raj1 |
| BD Pharmingen™ PE Mouse IgG2b κ Isotype Control | Wan et al., Sci Rep. 2021 Nov 11;11(1):22121 | BD Pharmingen | 555743 | 27-35 |
| PE/Cyanine7 Mouse IgG1, κ Isotype Ctrl Antibody | Lu et al., Cell. 2020 Mar 19;180(6):1081-1097.e24. | BioLegend | 400126 | MOPC-21 |
| BV421 Mouse IgG1, k Isotype Control | NA | BD Biosciences | 562438 | X40 |
| Brilliant Violet 605™ Mouse IgG2a, κ Isotype Ctrl Antibody | Tom et al., Mol Cell Biol. 2020 Mar 16;40(7):e00434-19 | BioLegend | 400270 | MOPC-173 |

- 1. **Cell lines**

| **Name** | **Citation** | **Supplier** | **Cat no.** | **Passage no.** | **Authentication test method** |
| --- | --- | --- | --- | --- | --- |
| NA |  |  |  |  |  |

- 1. **Organisms**

| **Name** | **Citation** | **Supplier** | **Strain** | **Sex** | **Age** | **Overall n number** |
| --- | --- | --- | --- | --- | --- | --- |
| NA |  |  |  |  |  |  |

- 1. **Sequence based reagents**

| **Name** | **Sequence** | **Supplier** |
| --- | --- | --- |
| NA |  |  |

- 1. **Biological samples**

| **Description** | **Source** | **Identifier** |
| --- | --- | --- |
| Liver tissue | Rejected transplant liver from Erasmus Medical Center | Hepatocyte donor 1 |
| Liver tissue | Patient from Erasmus Medical Center | Hepatocyte donor 2 |
| Liver tissue | Patient from Erasmus Medical Center | Hepatocyte donor 3 |
| Liver tissue | Patient from Erasmus Medical Center | Hepatocyte donor 4 |
| Liver tissue | Patient from Erasmus Medical Center | Hepatocyte donor 5 |
| Liver tissue | Patient from Erasmus Medical Center | Hepatocyte donor 6 |
| Tumor free liver tissue | Patient from Erasmus Medical Center | Hepatocyte donor 7 |
| Tumor free liver tissue | Patient from Erasmus Medical Center | Hepatocyte donor 8 |
| Tumor free liver tissue | Patient from Erasmus Medical Center | Hepatocyte donor 9 |
| Tumor free liver tissue | Patient from Erasmus Medical Center | Hepatocyte donor 10 |
| Tumor free liver tissue | Patient from Erasmus Medical Center | Hepatocyte donor 11 |
| Tumor free liver tissue | Patient from Erasmus Medical Center | Hepatocyte donor 12 |
| Tumor free liver tissue | Patient from Erasmus Medical Center | Hepatocyte donor 13 |
| Tumor free liver tissue | Patient from Erasmus Medical Center | Hepatocyte donor 14 |
| Tumor free liver tissue | Patient from Erasmus Medical Center | Hepatocyte donor 15 |
| HCC tumor tissue | Patient from Erasmus Medical Center | Hepatocyte donor 16 |
| Tumor free liver tissue | Patient from Erasmus Medical Center | Hepatocyte donor 16 |
| HCC tumor tissue | Patient from Erasmus Medical Center | Hepatocyte donor 17 |
| Tumor free liver tissue | Patient from Erasmus Medical Center | Hepatocyte donor 17 |
| HCC tumor tissue | Patient from Erasmus Medical Center | Hepatocyte donor 18 |
| Tumor free liver tissue | Patient from Erasmus Medical Center | Hepatocyte donor 18 |
| HCC tumor tissue | Patient from Erasmus Medical Center | Hepatocyte donor 19 |
| Tumor free liver tissue | Patient from Erasmus Medical Center | Hepatocyte donor 19 |
| HCC tumor tissue | Patient from Erasmus Medical Center | Hepatocyte donor 20 |
| Tumor free liver tissue | Patient from Erasmus Medical Center | Hepatocyte donor 20 |
| HCC tumor tissue | Patient from Erasmus Medical Center | Hepatocyte donor 21 |
| Tumor free liver tissue | Patient from Erasmus Medical Center | Hepatocyte donor 21 |
| HCC tumor tissue | Patient from Erasmus Medical Center | Hepatocyte donor 22 |
| Tumor free liver tissue | Patient from Erasmus Medical Center | Hepatocyte donor 22 |
| Peripheral blood | Patient from Erasmus Medical Center | Hepatocyte donor 18 |
| Peripheral blood | Patient from Erasmus Medical Center | Hepatocyte donor 22 |
| Buffy coat | Sanquin, the Netherlands | HBV Resolver 1 |
| Buffy coat | Sanquin, the Netherlands | HBV Resolver 2 |
| Buffy coat | Sanquin, the Netherlands | HBV Resolver 3 |
| Buffy coat | Sanquin, the Netherlands | HBV Resolver 4 |

- 1. **Deposited data**

| **Name of repository** | **Identifier** | **Link** |
| --- | --- | --- |
| PRIDE partner repository | PXD023143 | http://www.ebi.ac.uk/pride |

- 1. **Software**

| **Software name** | **Manufacturer** | **Version** |
| --- | --- | --- |
| GraphPad Prism | GraphPad Software, Inc | 8 |
| FlowJo | BD Biosciences | 10.6.1 |
| R | R core team | 4.0.0 |
| RStudio | RStudio | 1.0.44 |
| R-package GOplot1.0.2 | Walter et al. | 1.0.2 |
| R-package ggpubr0.4.0 | Alboukadel Kassambara | 0.4.0 |
| Adobe Illustrator | Adobe | 25.2 |
| Image Studio Lite | LI-COR | 5.2 |
| Peaks Studio | Bioinformatics Solutions Inc | 10.5 |

- 1. **Other (*e.g*. drugs, proteins, vectors etc.)**

| NA |  |  |
| --- | --- | --- |

- 1. **Please provide the details of the corresponding methods author for the manuscript:**

| Dr. Sonja I. Buschow, Department of Gastroenterology and Hepatology Erasmus MC, Doctor Molewaterplein 40, 3015 GD Rotterdam, the Netherlands; s.buschow@erasmusmc.nl |
| --- |

**2.0 Please confirm for randomised controlled trials all versions of the clinical protocol are included in the submission. These will be published online as supplementary information.**

| NA |
| --- |
